# A meta-analysis of pain intensity changes after placebo interventions in facial pain

**DOI:** 10.1097/PR9.0000000000001499

**Published:** 2026-10-05

**Authors:** Sabir Saluja, Ahed H. Kattaa, Bethany Shih, Mehma Jolly, Mensure Polat, Chris Antonio Lopez, Salaar Ahmed, Yuhao Huang, Paul D. McGeoch, QiLiang Chen, Alice Huai-Yu Li, Vafi Salmasi, Vivek P. Buch, Xiang Qian, Meredith Barad, Michael Lim, Sean Mackey, Ashwin G. Ramayya

**Affiliations:** a Department of Neurosurgery, Stanford University, Palo Alto, CA, USA; b Department of UCLA Institute for Society and Genetics, University of California Los Angeles, Los Angeles, CA, USA; c Department of Anesthesiology, Perioperative and Pain Medicine, Stanford University, Palo Alto, CA, USA; d Department of Neurology & Neurological Sciences, Stanford University, Palo Alto, CA, USA; e Department of Radiation Oncology, Stanford University, Stanford, CA, USA; f Department of Medicine (Oncology), Stanford University, Stanford, CA, USA; g Department of Otolaryngology - Head & Neck Surgery, Stanford University, Palo Alto, CA, USA

**Keywords:** Placebo effect, Facial pain, Meta-analysis

## Abstract

Interpreting pain improvements after interventions in clinical settings is challenging because placebo effects can influence patient-reported outcomes. In clinical practice, pain intensity reductions of 30% or greater are commonly considered indicative of meaningful analgesic benefit. However, the likelihood of achieving such reductions after placebo interventions is not known. We conducted a comprehensive meta-analysis to quantify pain intensity changes reported after placebo interventions in clinical trials of facial pain (FP). We systematically searched for randomized controlled trials (RCTs) and uncontrolled case series of FP treatments that included placebo or sham arms. The primary outcome was percentage change in pain intensity. The primary analysis focused on RCTs, with pooled effect sizes estimated using random-effects meta-analysis. Data from 115 studies (n = 3,208) were analyzed, including 71 RCTs (n = 2,128) and 44 uncontrolled case series (n = 1,080). Sham interventions yielded an average pain reduction of 29.73% (95% CI 24.09%-35.36%), with a prediction interval (−14.51% to 73.96%) among RCTs. Placebo responses varied by diagnosis (*P* < 0.001), with larger effects in temporomandibular disorders and classical trigeminal neuralgia. Cumulative probability analyses showed that ≥50% pain relief occurred in ∼25% of placebo-treated patients, whereas ≥75% improvement was reported in ∼2.5%. In FP, placebo interventions often produce mild to moderate relief but rarely yield dramatic improvements of ≥75%. These results can guide the interpretation of reported pain relief in clinical settings and inform clinical trial design in chronic pain conditions, suggesting that ≥75% pain intensity reductions after analgesic interventions are unlikely to be primarily driven by placebo effects.

## Abstract 2

Supplemental Digital Content is Available in the Text.

Placebo interventions in facial pain produce mild to moderate relief but rarely ≥75% improvement, suggesting ≥75% pain reductions after analgesic interventions are unlikely primarily placebo-driven.

## 1. Introduction

In clinical practice, interpreting patient-reported improvements in pain intensity after an intervention can be challenging due to the potential influence of the placebo effect. As randomization and blinding are often not feasible in clinical settings, a ≥30% reduction in pain intensity is commonly used as a threshold for a clinically meaningful response to an analgesic intervention, based on already established guidelines.^31,126^ This criterion is derived from psychometric studies that correlate reported changes in pain intensity after a placebo intervention. Placebo effects have been implicated in the mechanisms of and the treatments for a broad range of pain conditions, including idiopathic, nociceptive, and neuropathic pain.^11,12^ The mechanisms underlying placebo responses are thought to be complex and multifactorial, with evidence implicating both central nervous system sensitization and psychosocial factors in the phenomenon of subjective symptomatic improvement after nonpharmacologic or sham interventions for the treatment of pain. Notably, placebo response rates in neuropathic pain have increased substantially between 1982 and 2017, while those for pharmacological therapy have remained relatively stable over time.^39,118^

However, the magnitude of placebo effects in facial pain (FP) remains poorly characterized. Facial pain remains a significant cause of disability and discomfort to patients throughout the world, comprising 26% of cases of chronic pain by type.^11,12^ It is a heterogeneous condition with numerous etiologies and clinical presentations, making treatment challenging. There are numerous etiologies for FP, including neuropathic pain, musculoskeletal FP (eg, temporomandibular disorders [TMDs]), and persistent idiopathic (previously, “atypical”) FP. Various classification frameworks for FP conditions exist, including the *International Classification of Headache Disorders, third edition* (ICHD-3), and the Burchiel classification. Numerous treatment modalities are available to treat FP conditions arising from diverse etiologies.^8,16^ These include oral pharmacotherapy, local injections or nerve blocks, surgical procedures (eg, microvascular decompression, stereotactic ablation procedures, and central and peripheral neurostimulation), behavioral interventions, and alternative approaches, such as nonpharmacologic supplements and other complementary interventions. However, reports of these treatments have exhibited variable analgesic efficacy and clinical outcomes.

The existing literature suggests that placebo effects may play a significant role in patient-reported outcomes for FP interventions, given their recognized contribution to analgesic effects in treating chronic pain, and the challenges of implementing rigorous blinding and randomization in clinical settings.^20,40,118^ However, the magnitude of the placebo effect in FP treatments has not been systematically quantified. Establishing benchmarks for expected pain reduction after placebo interventions is crucial for guiding clinical decision-making and interpreting patient-reported outcomes.^31,36^

To this end, we conducted a comprehensive meta-analysis of placebo responses across published clinical trials of treatments for FP across a wide range of etiologies. We hypothesized a priori that placebo interventions would be associated with measurable but generally modest reductions in pain intensity, and that large improvements (eg, ≥75% pain reduction) would be uncommon. Accordingly, we aimed to describe the magnitude and rates of pain relief after placebo treatments. Our findings can serve as a valuable reference for clinicians and researchers, informing the design and interpretation of future studies and providing insight into improving the management of FP in clinical practice.^34,65,124^

## 2. Methods

### 2.1. Data sources and searches

The study was conducted in June 2024 in line with the Preferred Reporting Items for Systematic Reviews and Meta-Analyses guidelines.^109^ We selected clinical trials and case series that reported the results of pain interventions for FP. A comprehensive search strategy was developed, and predefined search strings were applied to 2 databases: PubMed/Medline and Google Scholar. The search terms were (“facial pain” OR “neuropathic facial pain” OR “trigeminal neuralgia” OR “trigeminal neuropathic pain” OR “trigeminal deafferentation pain” OR “postherpetic neuralgia” OR “temporomandibular pain” OR “atypical facial pain”) AND (“study” OR “trial” OR “evaluation” OR “series”). PROSPERO registration was not performed because data extraction had already been completed before awareness of the database.

### 2.2. Eligibility criteria

The inclusion criteria comprised studies published in English involving patients age 18 to 75 years with FP of any etiology that incorporated a placebo or sham intervention. Eligible study designs included randomized controlled trials (RCTs) as well as uncontrolled case series. Review articles, case reports, editorials, letters, or meta-analyses were excluded. Studies that reported no follow-up beyond the immediate post-treatment period and studies reporting an adjunct to the intervention rather than the intervention itself were excluded. After deduplication, 2 authors independently screened titles and abstracts, followed by full-text screening. Any conflicts were resolved by a third author. Data extraction was then performed independently by 2 authors (B.S. and M.J.) who manually reviewed each eligible study and recorded the data in a structured spreadsheet.

### 2.3. Data extraction

The primary outcome was the percent change in pain intensity in the placebo or sham arm, measured using the visual analogue scale or numerical rating scale. Percentage change from baseline was used to account for differences in pain assessment methods across studies. When multiple follow-up time points were reported, data from the latest follow-up were used.

In addition, the following variables were extracted from each study: (1) participant demographics, including age, sex, and number of subjects in the intervention and control arms; (2) category of intervention; (3) specific experimental and control interventions used; (4) FP subtype of study participants; (5) reported improvement in pain intensity; (6) estimates of variability (eg, SD or SE); (7) the duration between pain onset and intervention; and (8) follow-up duration between intervention and pain outcome assessment.

### 2.4. Statistical analysis

All measures of variability were standardized to 95% confidence intervals (CIs) to ensure consistency among reported pain outcomes. If the SEM was reported, the 95% CI was calculated using the formula: CI = mean ± (t* × SEM), where t* is the critical value from the t-distribution corresponding to n-1 degrees of freedom. In cases where the SD was reported, we estimated the SEM by dividing the SD by the square root of the sample size, and the same formula was used to compute the 95% CI.

For the primary meta-analysis, only randomized, double-blinded controlled trials with a placebo or sham arm were included to estimate placebo-related changes in pain intensity. Uncontrolled case series were not pooled with randomized trials but were evaluated separately in sensitivity analyses to assess the robustness of the findings. The probability of achieving different degrees of pain reduction was estimated (Table 1).

**Table 1:** Cumulative probability of percent pain improvement from empirical patient distribution.

% Improvement	Probability	Lower_95_CI	Upper_95_CI
−25	0.852	0.838	0.866
−20	0.852	0.838	0.866
−15	0.846	0.831	0.860
−10	0.846	0.831	0.860
−5	0.834	0.818	0.848
0	0.815	0.799	0.830
5	0.792	0.776	0.808
10	0.700	0.682	0.718
15	0.639	0.619	0.658
20	0.573	0.553	0.592
25	0.533	0.513	0.553
30	0.509	0.489	0.529
35	0.390	0.371	0.410
40	0.344	0.325	0.363
45	0.340	0.321	0.359
50	0.259	0.242	0.277
55	0.201	0.185	0.217
60	0.053	0.045	0.063
65	0.041	0.034	0.050
70	0.037	0.030	0.045
75	0.026	0.020	0.033
80	0.000	0.000	0.002
85	0.000	0.000	0.002
90	0.000	0.000	0.002
95	0.000	0.000	0.002
100	0.000	0.000	0.002

CI, confidence interval.

To calculate a population-level estimate of pain changes in placebo interventions, a random-effects model was generated using RStudio and the “meta” library, with the Hartung–Knapp adjustment to provide more conservative estimates of treatment effects.^9,53,57,89^ The restricted maximum-likelihood estimator was used for calculating between-study variance (τ^2^), as this method provides a balance between unbiased estimation and optimal statistical properties.^122^ Heterogeneity was quantified using multiple complementary approaches: *I*^2^ statistics (with values of 25%, 50%, and 75% indicating low, moderate, and high heterogeneity, respectively), H^2^ statistics (ratio of total variability to sampling variability), and Cochran's *Q* test. The Q-Profile method was used to calculate CIs for τ^2^ and τ statistics.

To evaluate the potential influence of regression-to-the-mean (RTM) on observed placebo responses, a meta-regression analysis was performed examining the association between baseline pain intensity and percentage change in pain scores across RCT studies. In addition, a sensitivity analysis was conducted, restricting the data set to studies reporting ≥2 baseline pain measurements before the intervention, as repeated baseline assessments can help mitigate RTM effects. Publication bias was evaluated to examine whether small, underpowered trials may have exaggerated the observed placebo response. A funnel plot was visually inspected, and Egger's regression-based test was conducted. A trim-and-fill analysis was performed to estimate the potential impact of missing studies on the overall effect size. In addition, a random-effects meta-regression model using the restricted-maximum likelihood estimator was conducted to investigate whether the duration of pain before intervention was associated with the magnitude of the placebo-associated analgesic effect across the included studies.

### 2.5. Subgroup analysis

Prespecified subgroup analyses were conducted to explore effect size variations across (1) pain types: atypical facial pain (AFP, or persistent idiopathic facial pain), classical trigeminal neuralgia (TN), TMDs, and trigeminal neuropathic pain; and (2) intervention types: medication, procedural (eg, radiofrequency ablation), alternative therapies (eg, homeopathy, acupuncture), and noninvasive neuromodulation (eg, transcranial magnetic stimulation). In each subgroup analysis, between-group heterogeneity was assessed using the *Q*-test for subgroup differences, and within-group heterogeneity was evaluated using *I*^2^ statistics.

The authors acknowledge that the term *atypical facial pain* has been replaced by *persistent idiopathic facial pain* in the ICHD-3. However, to ensure comprehensive capture of the literature, the term *atypical facial pain* was retained in this study because it remains commonly used in the neurosurgical literature, particularly within the Burchiel classification. Similarly, the term *trigeminal neuropathic pain*, as defined in the Burchiel classification, was used in place of *painful trigeminal neuropathy* as defined in ICHD-3.

## 3. Results

The literature search yielded 115 eligible studies,^1–7,10,13–15,17–19,21–30,32,33,35,37,38,41–52,54–56,58–64,66–88,90–108,110–117,119–121,123,125,127–136^ including a total of 3,208 patients with FP who received placebo or sham interventions (Fig. 1). Of these, 71 were RCTs (n = 2,128), and 44 were uncontrolled case series (n = 1,080). The complete list of included studies is provided in Supplemental Tables 1 and 2 (available at https://links.lww.com/PR9/A446). Regarding pain conditions among RCT studies, there were 6 studies on AFP, 17 studies on classical TN, 43 studies on TMD, and 5 studies on trigeminal neuropathic pain.

**Figure 1.**
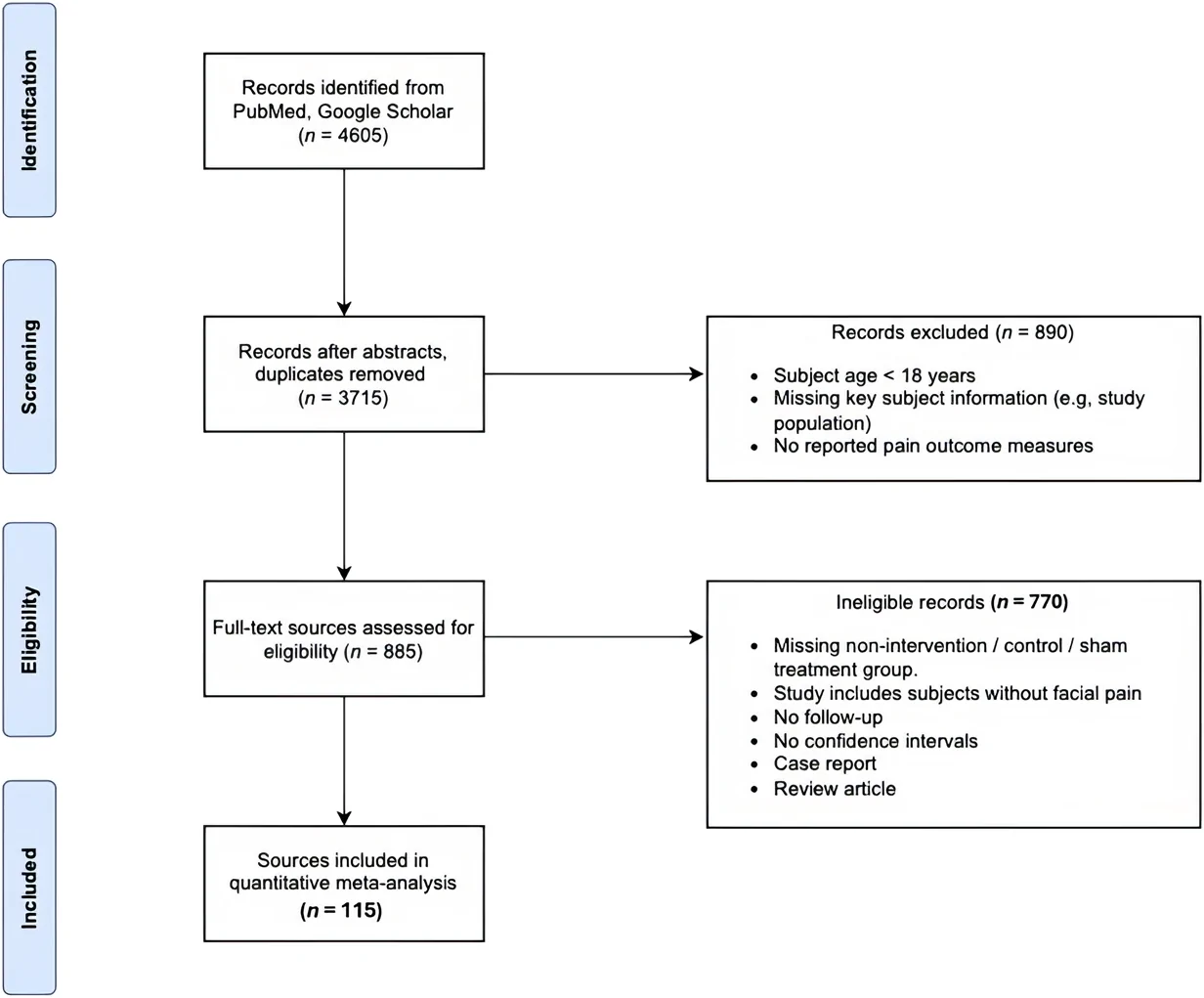
PRISMA 2020 flow diagram for source search. Visual graphic of search selection showing each step and reasons for exclusion of studies from the analysis. PRISMA, Preferred Reporting Items for Systematic Reviews and Meta-analyses.

Across RCT studies, the pooled effect size was 29.73% pain reduction with placebo/sham (95% CI 24.09%–35.36), with very high heterogeneity (*I*^2^ = 100.0%) (Supplementary Fig. 1, available at https://links.lww.com/PR9/A446). The corresponding prediction interval for this estimate was −14.51% to 73.96% pain improvement with placebo/sham interventions. The data were transformed using a cumulative probability distribution curve. We found that there was a ∼50% likelihood of observing at least 30% change in pain after a placebo intervention, a ∼25% likelihood of observing at least 50% change in pain intensity, a ∼2.5% likelihood of observing at least 75% change in pain intensity, and a ∼0% likelihood of observing at least 80% change in pain intensity (Fig. 2A–C).

**Figure 2.**
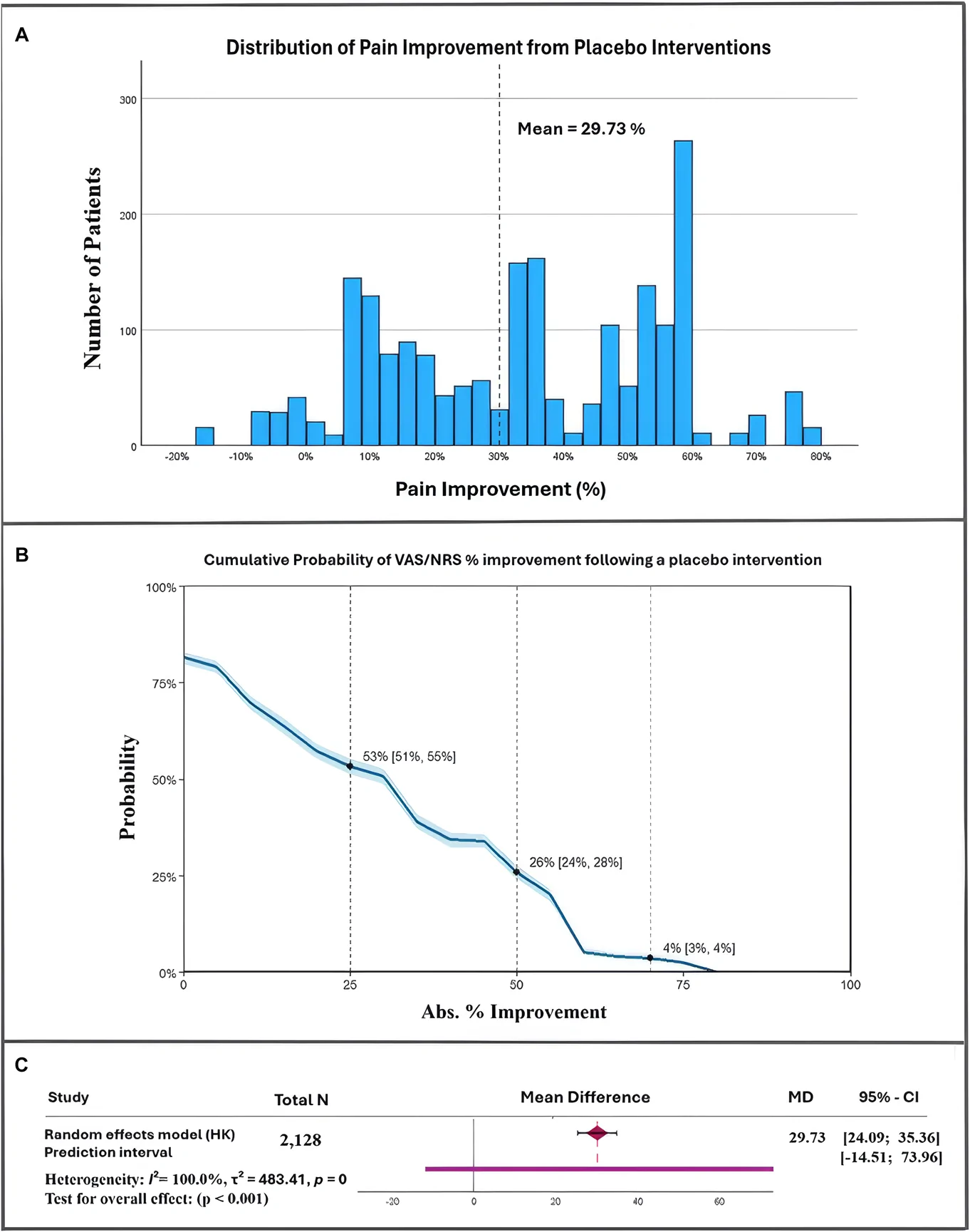
(A) Distribution of percent improvement shown in a histogram for all included patients. (B) Distribution of percent improvement for all included patients transformed to cumulative probability distribution, where the *y*-value is the probability of occurrence of pain improvement greater than or equal to the corresponding value on the *x*-axis. Curve from the empirical distribution of patient responses aggregated is displayed. (C) Summary of results from random-effects analysis, with estimated MD, 95% CI of MD, and estimated prediction interval for percent improvement for a facial pain intervention for an individual patient. CI, confidence interval; MD, mean difference; NRS, numerical rating scale; VAS, visual analogue scale.

In the pain type stratification analyses (Fig. 3A), classical TN showed the greatest pain improvement in placebo interventions (37.86%; 95% CI 26.37–49.35), followed by TMD (32.62%; 25.62–39.62), followed by trigeminal neuropathic pain (11.82%; 8.37–15.27), and finally AFP (3.24%; −2.75 to 9.22). The test for subgroup differences was highly significant (*Q* = 57.57, *df* = 3, *P* < 0.001), indicating that diagnosis is a significant moderator of placebo effects. When stratified by intervention type (Fig. 3B), pain improvement in the procedure subgroup was found to be 40.11% (11.66–68.54), followed by 31.84% (24.31–39.38) for oral medications, 29.62% (10.73–48.49) for alternative treatments, 27.48% (13.54–41.41) for noninvasive neuromodulation, and 20.94% (7.15–34.73) in injections or nerve blocks. However, these differences were not statistically significant (*Q* = 2.47, *df* = 4, *P* = 0.65). Characteristics of placebo/sham procedures in the surgical and injection/nerve block categories are summarized in Supplemental Table 3 (available at https://links.lww.com/PR9/A446), which provides details regarding the administration of placebo interventions across these study types.

**Figure 3.**
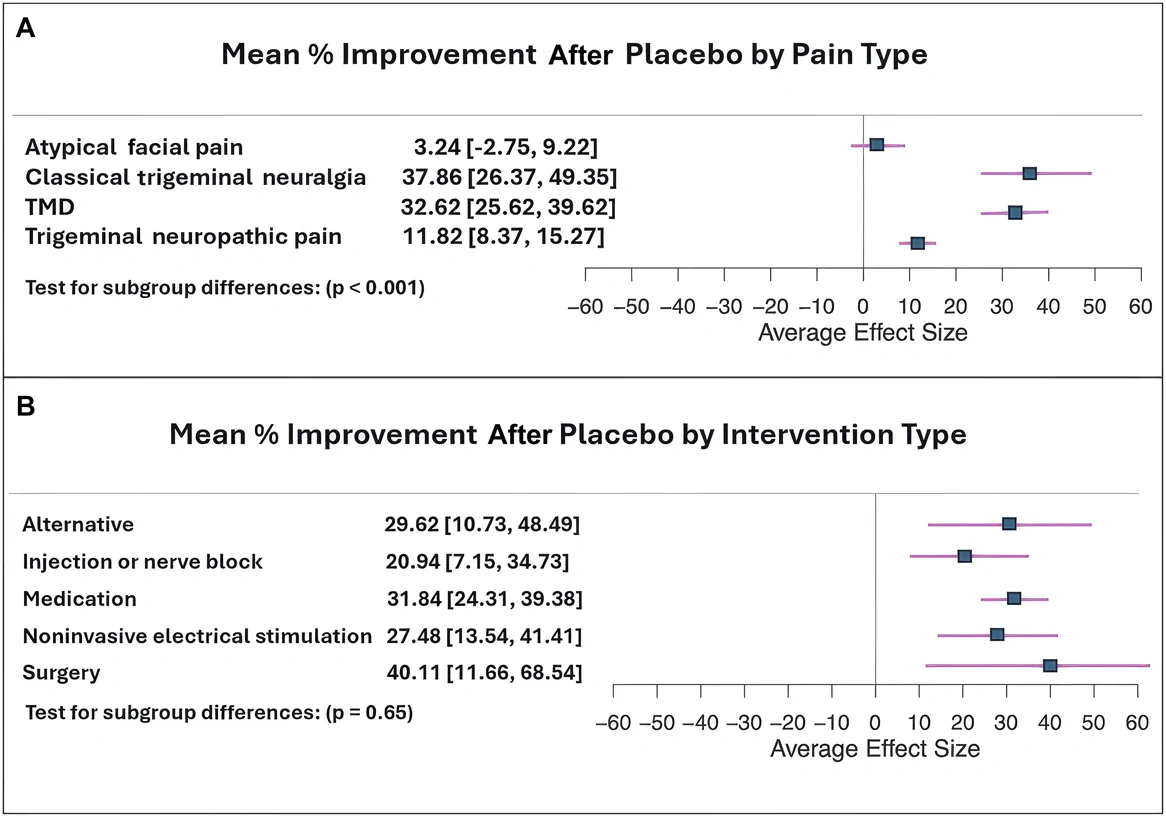
Subgroup meta-analysis results. Percent changes in pain improvement and 95% confidence intervals computed by random-effects analysis in subgroups stratified by (A) pain type and (B) intervention type. TMD, temporomandibular disorder.

Meta-regression analysis showed no statistically significant association between baseline pain intensity and percentage change in pain (Wald χ^2^ = 2.02, *df* = 1, *P* = 0.155), with baseline pain intensity explaining only 2.9% of the variability in percentage pain reduction. This suggests that differences in baseline pain severity do not meaningfully predict the magnitude of pain improvement observed across studies. Residual heterogeneity remained substantial (*I*^2^ = 99.9%, τ^2^ = 471.82), suggesting that variability in effect sizes is largely driven by between-study differences rather than sampling error alone. These findings suggest that regression to the mean related to baseline severity does not appear to be a major contributor to the observed pain reductions.

A sensitivity analysis was conducted, including only studies that reported ≥2 baseline pain measurements, and showed no significant association between baseline pain intensity and percentage change in pain (Wald χ^2^ = 1.15, *df* = 1, *P* = 0.283), with baseline pain explaining only 1.0% of the variability in effect sizes.

Visual inspection of the funnel plot did not indicate asymmetry (Fig. 4). Egger's test showed no evidence of small-study effects (*P* = 0.985), suggesting that effect sizes were not significantly related to study precision. The trim-and-fill analysis indicated that no studies required imputation, and the adjusted overall effect size remained unchanged (29.73, 95% CI 24.10–35.37). These findings suggest that publication bias is unlikely to have meaningfully influenced the observed placebo effect (small, underpowered studies are not skewing the overall effect size).

**Figure 4.**
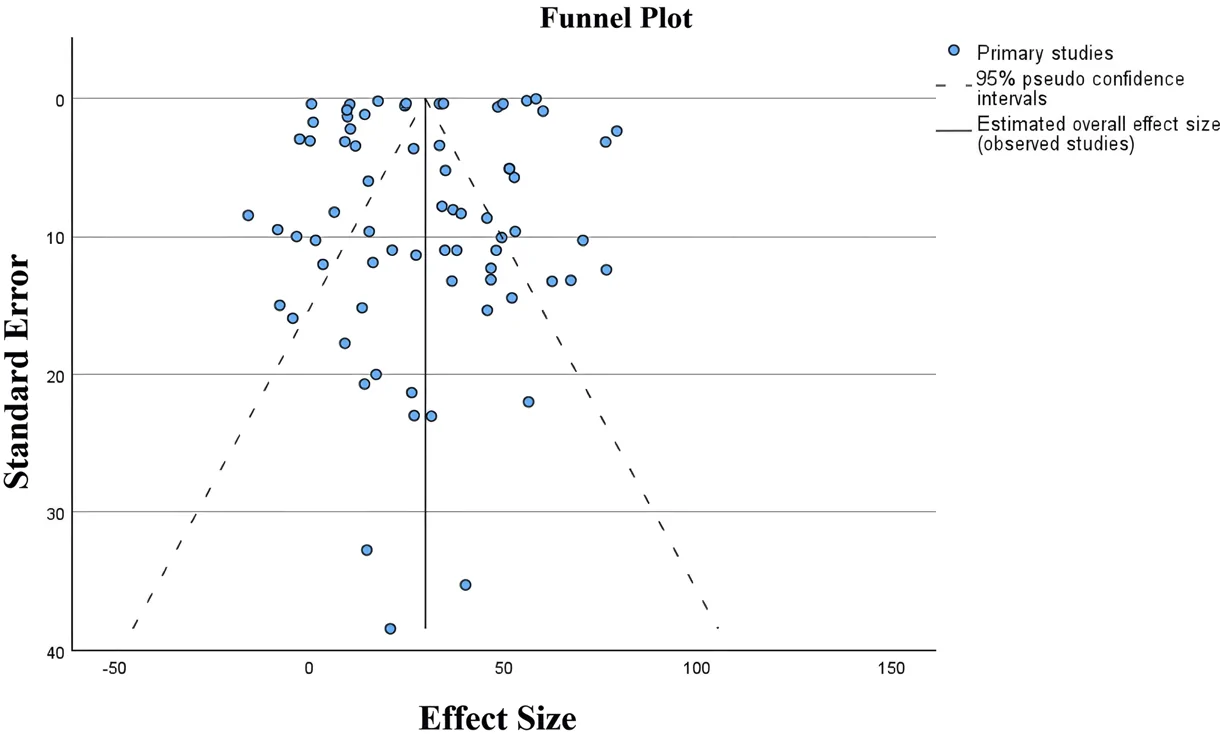
Funnel plot assessing publication bias. Each point represents an individual study plotted by effect size and standard error. The vertical line indicates the pooled effect size and the dashed lines represent the 95% pseudo confidence intervals. The symmetric distribution of studies suggests no evidence of publication bias or small-study effects.

To assess the robustness of the findings, a sensitivity analysis was conducted by adding 44 uncontrolled case series (n = 1,080) to the 71 randomized, double-blinded controlled trials (n = 2,128). When combined, the overall estimated placebo response was slightly lower at 28.85% (95% CI 22.02%–35.69%). The minimal change in effect size when adding nonrandomized studies indicates that the observed placebo response is largely consistent across study designs, supporting the robustness of the primary RCT-based estimate.

Information regarding concomitant treatments was extracted from the included studies. Several studies prohibited the use of concomitant pharmacological or pain-related therapies during the trial. In studies where concomitant treatments were permitted, participants were allowed to continue stable background medications as needed; however, no study reported escalation of medication doses or initiation of new analgesic or interventional therapies during the study period.

Among studies reporting the duration of pain before intervention, the association between pain duration and the magnitude of the placebo-associated analgesic effect was evaluated using meta-regression. The dependent variable was the study-specific effect size, and mean pain duration was included as a continuous moderator. A total of 23 studies contributed data to this analysis. The meta-regression showed that pain duration was not a statistically significant predictor of effect size (QM = 0.949, *P* = 0.330). The estimated regression coefficient was negative (β = −0.428), indicating a slight tendency toward smaller placebo-associated analgesic effects with increasing pain duration. However, the 95% CI included zero (95% CI −1.288 to 0.433), indicating that this relationship was not statistically supported.

The residual heterogeneity remained extremely high after including pain duration in the model (τ^2^ = 569.80; *I*^2^ = 99.80%), and the moderator explained virtually none of the between-study heterogeneity (*R*^2^ = 0.0%). The test for residual heterogeneity was highly significant (QE = 3703.57, *df* = 21, *P* < 0.001), indicating that substantial variability among studies remained unexplained by pain duration alone. These findings suggest that the duration of pain before intervention was not associated with the magnitude of the placebo-associated analgesic effect in the included studies. Consequently, pain duration does not appear to explain the considerable between-study heterogeneity observed in this meta-analysis.

## 4. Discussion

In this study, we performed a meta-analysis of placebo effects in treatments for FP. Although previous meta-analyses and reviews have quantified the placebo effect in treatments for various medical conditions,^43,87,120,131^ to our knowledge, this is the first meta-analysis estimating the effect size of the placebo effects across different treatment types specifically for patients with FP.

Using a pooled sample of 2,128 patients from 71 placebo-randomized controlled studies, we quantified the reported changes in pain intensity in response to interventions for FP and showed that placebo/sham treatments are associated with an average pain reduction of approximately 29.73% with a prediction interval of −14.51% to 73.96%. Our estimated likelihood function further demonstrated that approximately 25% of patients experience >50% pain reduction with placebo treatment, whereas virtually no patients experience >75% reduction. These findings suggest that reports of >75% pain reduction are unlikely to be primarily attributable to placebo effects. Importantly, our data do not imply that all reports of pain improvement below 50% are placebo-mediated; rather, such outcomes remain compatible with placebo-driven analgesic responses. Subgroup analyses further indicated that diagnosis significantly moderates placebo response, with classical TN showing the greatest improvement, followed by TMD, trigeminal neuropathic pain, and AFP. This variability may reflect differences in underlying pathophysiology, symptom fluctuation, or patient expectations across FP conditions. The influence of concomitant therapies is unlikely to have substantially contributed to the observed placebo-associated analgesic effects, given that studies permitting continuation of background treatments did not report escalation of medication dosages or initiation of new analgesic or interventional therapies during the study period. In addition, differences in disease course, symptom patterns, and spontaneous fluctuations in pain severity across FP conditions may contribute to the observed variation in placebo-associated analgesic responses. Further longitudinal studies evaluating the natural history of individual FP disorders are needed to better understand these potential influences.

Notably, although RTM can contribute to apparent symptom improvement in pain trials, our meta-regression analysis found no significant association between baseline pain intensity and the magnitude of pain reduction, and baseline pain explained only a very small proportion of the variability in effect sizes. Sensitivity analyses restricted to studies reporting multiple baseline pain measurements yielded similar findings. Together, these results suggest that RTM related to baseline pain severity is unlikely to be a major contributor to the observed placebo-related pain reductions.

These results have important implications for researchers and clinicians who study and manage patients with chronic pain. It is common practice for patients to undergo trial therapies with medical devices before permanent implantation to predict the likelihood of a successful response. Our findings suggest that using a higher threshold for pain improvement, such as 75%, may improve the predictive ability of trial devices for chronic pain. Our finding that larger reported pain improvements are less likely to reflect a placebo effect aligns with U.S. Preventive Services Task Force guidelines stating that “dramatic results in uncontrolled experiments” constitute higher-quality evidence than descriptive case series.^53^ As such, clinicians should consider such large effects when evaluating therapeutic efficacy.

Several important limitations should be considered when interpreting the findings of the study. First, there was significant heterogeneity (*I*^2^ = 100%), which reflects the broad inclusion criteria for study selection that were used in our meta-analysis. Although this approach enabled us to generate a comprehensive overview of placebo effects in FP, it also increased the heterogeneity of our data, resulting in noisier estimates. Second, limited reporting of follow-up duration in many included studies constrained our ability to characterize the temporal dynamics of placebo responses. Studies with short follow-up periods may overestimate placebo effects by capturing early improvements, whereas longer follow-up periods may fail to reflect the full trajectory of treatment response if benefits diminish over time. Third, as with most conventional meta-analyses, our analysis relied on aggregate study-level rather than individual patient-level data because individual participant data were not available for the included studies. Consequently, we were unable to evaluate interindividual variability or identify patient-level moderators of placebo response, including prior healthcare experiences and psychosocial factors. Our findings should therefore be interpreted as population-level benchmarks rather than predictors of placebo response for individual patients. Future individual-patient data meta-analyses would allow for more precise characterization of patient-level variability and determinants of the placebo response. Moreover, there was an uneven distribution of intervention types. The current study could identify only 4 studies assessing surgical procedures with a placebo or sham arm. This reflects the broader scarcity of sham-controlled surgical trials in FP and limits the strength of conclusions regarding placebo effects in surgical interventions. Despite these limitations, our study provides an important initial benchmark for estimating the magnitude of placebo responses in FP across a heterogeneous patient population.

Future research should extend this work by examining placebo response thresholds across a broader range of pain conditions to determine whether the patterns observed in FP generalize to other chronic pain syndromes. Such efforts would benefit from standardized reporting of pain outcomes and consistent follow-up intervals across trials. Establishing prospective registries incorporating patient-level data from randomized placebo-controlled studies would further improve understanding of the persistence and variability of placebo responses over time. These data sets would also enable evaluation of placebo effects across multidimensional domains of pain beyond intensity alone, including pain interference, fatigue, physical function, and mood. Collectively, these directions may support the development of more refined frameworks for distinguishing placebo-mediated improvement from true therapeutic response and ultimately improve the evaluation and management of chronic pain.

## 5. Conclusions

In conclusion, this comprehensive meta-analysis provides the first quantitative benchmark for placebo responses in FP. By pooling data from 71 studies, we found that sham interventions yield moderate pain relief on average but rarely produce dramatic improvements of ≥75%. These findings can inform the interpretation of uncontrolled studies and guide clinical trial design in FP and other chronic pain conditions. Using a 75% pain reduction threshold to define patient responders may help mitigate placebo effects and identify robust, clinically meaningful treatment outcomes. However, we recognize that this represents a stringent benchmark, which may be difficult for many therapies to achieve and may have important implications for how treatment efficacy is interpreted.

## Disclosures

**Declaration of competing interest:** The authors declare the following financial interests/personal relationships which may be considered as potential competing interests: **QiLiang Chen:** Funding from VA CDA2 (1IK2BX006567). **Michael Lim:** Funding from Biohaven, Arbor, Accuray; Consultant: Biohaven, GCAR, CraniUS, Insightec, Mediflix, Merck, Neumeous, Novocure, S1 Oncology, Servier, Stryker, Hemispherian, Hoth, Noxxon, Sanianoia, VBI; Data Safety Monitoring Board participation: Cellebrain, OmixAI; Leadership role: Egret Therapeutics; Shareholder: Cellebrain, Erget Therapeutics, OmixAI. **Sean Mackey:** Funding from Science and Innovation FDA, Patient-Centered Outcomes Research Institute; NIH/NINDS, National Institutes of Health, University of Oklahoma/National Institutes of Health; Royalties/licenses: McGraw Hill LLC; Payment/Honoraria: American Society of Anesthesiologists (ASA) Annual Meeting, American Academy of Pain Medicine (AAPM) 2025 Annual Meeting, Pain Societies of the Carolinas Annual Meeting (PSC), University of Texas, MD Anderson Cancer Center Translational Pain Conference, University of Arizona (UACOM) Medical Scientist Training Program (MSTP) Colloquium, Visiting Professor at Vanderbilt, New York State Pain Society Annual Meeting & Scientific Sessions, North American Neuromodulation Society (NANS) 26^th^ Annual Meeting, Canadian Pain Society (CPS), International Pelvic Pain Society (IPPS), International Association for the Study of Pain (IASP), World Institute of Pain (WIP), Dalhousie University, American Australian Association Research Exchange, Association of University Anesthesiologists (AUA), American Academy of Orofacial Pain Scientific Meeting 2026, Endeavor Health’s First Annual Congress for Pain Care Advancement, Quinn Johnston | Lee v SBLHS, NIH, National Academies. The remaining authors declare no conflicts of interest.

## Supplemental digital content

Supplemental digital content associated with this article can be found online at https://links.lww.com/PR9/A446.

## Supplementary Material

## References

[1] Agius AM Jones NS Muscat R. A randomized controlled trial comparing the efficacy of low-dose amitriptyline, amitriptyline with pindolol and surrogate placebo in the treatment of chronic tension-type facial pain. Rhinology 2013;51:143–53. 10.4193/Rhino12.187 23671895

[2] al Balawi S Tariq M Feinmann C. A double-blind, placebo-controlled, crossover, study to evaluate the efficacy of subcutaneous sumatriptan in the treatment of atypical facial pain. Int J Neurosci 1996;86:301–9. 10.3109/00207459608986720 8884400

[3] Alencar FGP Viana PG Zamperini C Becker A. Patient education and self-care for the management of jaw pain upon awakening: a randomized controlled clinical trial comparing the effectiveness of adding pharmacologic treatment with cyclobenzaprine or tizanidine. J Oral Facial Pain Headache 2014;28:119–27. 10.11607/ofph.963 24822235

[4] Amanat D Ebrahimi H Lavaee F Alipour A. The adjunct therapeutic effect of lasers with medication in the management of orofacial pain: double blind randomized controlled trial. Photomed Laser Surg 2013;31:474–9. 10.1089/pho.2013.3555 24102165

[5] Antonio C Zurek J Creighton P Johnson K Heard C. Reducing the pain of intranasal drug administration. Pediatr Dent 2011;33:415–9. 22104710

[6] Arantes GM Arantes VMN Ashmawi HA Posso IP. Tenoxicam controls pain without altering orthodontic movement of maxillary canines. Orthod Craniofac Res 2009;12:14–9. 10.1111/j.1601-6343.2008.01432.x 19154270

[7] Arikan OK Birol A Tuncez F Erkek E Koc C. A prospective randomized controlled trial to determine if cryotherapy can reduce the pain of patients with minor form of recurrent aphthous stomatitis. Oral Surg Oral Med Oral Pathol Oral Radiol Endod 2006;101:e1–5. 10.1016/j.tripleo.2005.07.035 16360599

[8] Baad-Hansen L Benoliel R. Neuropathic orofacial pain: facts and fiction. Cephalalgia 2017;37:670–9. 10.1177/0333102417706310 28403646

[9] Balduzzi S Rücker G Schwarzer G. How to perform a meta-analysis with R: a practical tutorial. Evid Based Mental Health 2019;22:153–60. 10.1136/ebmental-2019-300117 PMC10231495 31563865

[10] Barbosa MA Tahara AK Ferreira IC Intelangelo L Barbosa AC. Effects of 8 weeks of masticatory muscles focused endurance exercises on women with oro-facial pain and temporomandibular disorders: a placebo randomised controlled trial. J Oral Rehabil 2019;46:885–94. 10.1111/joor.12823 31099036

[11] Beecher HK. The powerful placebo. JAMA 1955;159:1602. 10.1001/jama.1955.02960340022006 13271123

[12] Benedetti F. Placebo effects. 2nd ed. Oxford: Oxford University Press, 2014.

[13] Bertolami CN Gay T Clark GT Rendell J Shetty V Liu C Swann DA. Use of sodium hyaluronate in treating temporomandibular joint disorders: a randomized, double-blind, placebo-controlled clinical trial. J Oral Maxillofac Surg 1993;51:232–42. 10.1016/s0278-2391(10)80163-6 8445463

[14] Bisla S Gupta A Agarwal S Singh H Sehrawat A Singh A. Evaluation of transcutaneous electrical nerve stimulation as an adjunct therapy in trigeminal neuralgia: a randomized double-blind placebo-controlled clinical study. J Dent Anesth Pain Med 2021;21:565–74. 10.17245/jdapm.2021.21.6.565 PMC8637911 34909474

[15] Bittar GT Graff-Radford SB. The effects of streptomycin/lidocaine block on trigeminal neuralgia: a double blind crossover placebo controlled study. Headache 1993;33:155–60. 10.1111/j.1526-4610.1993.hed3303155.x 8486515

[16] Burchiel KJ. A new classification for facial pain. Neurosurgery 2003;53:1164–7. 10.1227/01.neu.0000088806.11659.d8 14580284

[17] Cahlin BJ Dahlström L. No effect of glucosamine sulfate on osteoarthritis in the temporomandibular joints: a randomized, controlled, short-term study. Oral Surg Oral Med Oral Pathol Oral Radiol Endod 2011;112:760–6. 10.1016/j.tripleo.2011.06.012 22001199

[18] Cetiner S Kahraman SA Yücetaş S. Evaluation of low-level laser therapy in the treatment of temporomandibular disorders. Photomed Laser Surg 2006;24:637–41. 10.1089/pho.2006.24.637 17069496

[19] Chiu WK Cheung LK. Efficacy of preoperative oral rofecoxib in pain control for third molar surgery. Oral Surg Oral Med Oral Pathol Oral Radiol Endod 2005;99:e47–53. 10.1016/j.tripleo.2005.02.075 15897845

[20] Colloca L Barsky AJ. Placebo and nocebo effects. N Engl J Med 2020;382:554–61. 10.1056/NEJMra1907805 32023375

[21] Curtis P Gartman LA Green DB. Utilization of ketorolac tromethamine for control of severe odontogenic pain. J Endod 1994;20:457–9. 10.1016/S0099-2399(06)80038-5 7996118

[22] da Cunha LA Firoozmand LM da Silva AP Camargo SEA Oliveira W. Efficacy of low-level laser therapy in the treatment of temporomandibular disorder. Int Dent J 2008;58:213–7. 10.1111/j.1875-595x.2008.tb00351.x 18783114

[23] da Silva MAMR Botelho AL Turim CV da Silva AMBR. Low level laser therapy as an adjunctive technique in the management of temporomandibular disorders. Cranio 2012;30:264–71. 10.1179/crn.2012.040 23156967

[24] De Icco R Putortì A De Paoli I Ferrara E Cremascoli R Terzaghi M Toscano G Allena M Martinelli D Cosentino G Grillo V Colagiorgio P Versino M Manni R Sances G Sandrini G Tassorelli C. Anodal transcranial direct current stimulation in chronic migraine and medication overuse headache: a pilot double-blind randomized sham-controlled trial. Clin Neurophysiol 2021;132:126–36. 10.1016/j.clinph.2020.10.014 33271482

[25] Demirkol N Sari F Bulbul M Demirkol M Simsek I Usumez A. Effectiveness of occlusal splints and low-level laser therapy on myofascial pain. Lasers Med Sci 2015;30:1007–12. 10.1007/s10103-014-1522-7 24504660

[26] DeNucci DJ Sobiski C Dionne RA. Triazolam improves sleep but fails to alter pain in TMD patients. J Orofac Pain 1998;12:116–23. 9656889

[27] DeVocht JW Goertz CM Hondras MA Long CR Schaeffer W Thomann L Spector M Stanford CM. A pilot study of a chiropractic intervention for management of chronic myofascial temporomandibular disorder. J Am Dent Assoc 2013;144:1154–63. 10.14219/jada.archive.2013.0034 PMC4103021 24080932

[28] Dias WCFGDS Cavalcanti RVA Magalhães Júnior HV Pernambuco LDA Alves GÂDS. Effects of photobiomodulation combined with orofacial myofunctional therapy on the quality of life of individuals with temporomandibular disorder. Codas 2022;34:e20200313. 10.1590/2317-1782/20212020313 PMC9886175 35416889

[29] Diener HC Marmura MJ Tepper SJ Cowan R Starling AJ Diamond ML Hirman J Mehta L Brevig T Sperling B Cady R. Efficacy, tolerability, and safety of eptinezumab in patients with a dual diagnosis of chronic migraine and medication-overuse headache: subgroup analysis of PROMISE-2. Headache 2021;61:125–36. 10.1111/head.14036 33314079

[30] Dolwick MF Diaz D Freburg-Hoffmeister DL Widmer CG. A randomized, double-blind, placebo-controlled study of the efficacy of steroid supplementation after temporomandibular joint arthrocentesis. J Oral Maxillofac Surg 2020;78:1088–99. 10.1016/j.joms.2020.02.022 32201122

[31] Dworkin RH Turk DC Wyrwich KW Beaton D Cleeland CS Farrar JT Haythornthwaite JA Jensen MP Kerns RD Ader DN Brandenburg N Burke LB Cella D Chandler J Cowan P Dimitrova R Dionne R Hertz S Jadad AR Katz NP Kehlet H Kramer LD Manning DC McCormick C McDermott MP McQuay HJ Patel S Porter L Quessy S Rappaport BA Rauschkolb C Revicki DA Rothman M Schmader KE Stacey BR Stauffer JW Von Stein T White RE Witter J Zavisic S. Interpreting the clinical importance of treatment outcomes in chronic pain clinical trials: IMMPACT recommendations. J Pain 2008;9:105–21. 10.1016/j.jpain.2007.09.005 18055266

[32] Ebrahimi H Najafi S Khayamzadeh M Zahedi A Mahdavi A. Therapeutic and analgesic efficacy of laser in conjunction with pharmaceutical therapy for trigeminal neuralgia. J Lasers Med Sci 2018;9:63–8. 10.15171/jlms.2018.13 PMC5775958 29399314

[33] Emshoff R Bösch R Pümpel E Schöning H Strobl H. Low-level laser therapy for treatment of temporomandibular joint pain: a double-blind and placebo-controlled trial. Oral Surg Oral Med Oral Pathol Oral Radiol Endod 2008;105:452–6. 10.1016/j.tripleo.2007.09.012 18329580

[34] Enck P Bingel U Schedlowski M Rief W. The placebo response in medicine: minimize, maximize or personalize? Nat Rev Drug Discov 2013;12:191–204. 10.1038/nrd3923 23449306

[35] Eslamian L Borzabadi-Farahani A Edini HZ Badiee MR Lynch E Mortazavi A. The analgesic effect of benzocaine mucoadhesive patches on orthodontic pain caused by elastomeric separators, a preliminary study. Acta Odontol Scand 2013;71:1168–73. 10.3109/00016357.2012.757358 23301559

[36] Farrar JT Young JP LaMoreaux L Werth JL Poole MR. Clinical importance of changes in chronic pain intensity measured on an 11-point numerical pain rating scale. PAIN 2001;94:149–58. 10.1016/S0304-3959(01)00349-9 11690728

[37] Feinmann C Harris M Cawley R. Psychogenic facial pain: presentation and treatment. Br Med J (Clin Res Ed) 1984;288:436–8. 10.1136/bmj.288.6415.436 PMC1444752 6419955

[38] Ferreira APDL Costa DRAD Oliveira AISD Carvalho EAN Conti PCR Costa YM Bonjardim LR. Short-term transcutaneous electrical nerve stimulation reduces pain and improves the masticatory muscle activity in temporomandibular disorder patients: a randomized controlled trial. J Appl Oral Sci 2017;25:112–20. 10.1590/1678-77572016-0173 PMC5393531 28403351

[39] Finnerup NB Haroutounian S Baron R Dworkin RH Gilron I Haanpaa M Jensen TS Kamerman PR McNicol E Moore A Raja SN Andersen NT Sena ES Smith BH Rice ASC Attal N. Neuropathic pain clinical trials: factors associated with decreases in estimated drug efficacy. PAIN 2018;159:2339–46. 10.1097/j.pain.0000000000001340 PMC6193835 30015707

[40] Finniss DG Kaptchuk TJ Miller F Benedetti F. Biological, clinical, and ethical advances of placebo effects. Lancet 2010;375:686–95. 10.1016/S0140-6736(09)61706-2 PMC2832199 20171404

[41] Forssell H Tasmuth T Tenovuo O Hampf G Kalso E. Venlafaxine in the treatment of atypical facial pain: a randomized controlled trial. J Orofac Pain 2004;18:131–7. 15250433

[42] Fricová J Klírová M Masopust V Novák T Vérebová K Rokyta R. Repetitive transcranial magnetic stimulation in the treatment of chronic orofacial pain. Physiol Res 2013;62:S125–34. 10.33549/physiolres.932575 24329692

[43] Gao J Zhao C Jiang W Zheng B He Y. Effect of acupuncture on cognitive function and quality of life in patients with idiopathic trigeminal neuralgia. J Nerv Ment Dis 2019;207:171–4. 10.1097/NMD.0000000000000937 30720599

[44] Gazerani P Pedersen NS Staahl C Drewes AM Arendt-Nielsen L. Subcutaneous Botulinum toxin type A reduces capsaicin-induced trigeminal pain and vasomotor reactions in human skin. PAIN 2009;141:60–9. 10.1016/j.pain.2008.10.005 19004549

[45] Gerschman JA Ruben J Gebart-Eaglemont J. Low level laser therapy for dentinal tooth hypersensitivity. Aust Dent J 1994;39:353–7. 10.1111/j.1834-7819.1994.tb03105.x 7832682

[46] Goddard G Karibe H McNeill C Villafuerte E. Acupuncture and sham acupuncture reduce muscle pain in myofascial pain patients. J Orofac Pain 2002;16:71–6. 11889662

[47] Gremeau-Richard C Woda A Navez ML Attal N Bouhassira D Gagnieu MC Laluque JF Picard P Pionchon P Tubert S. Topical clonazepam in stomatodynia: a randomised placebo-controlled study. PAIN 2004;108:51–7. 10.1016/j.pain.2003.12.002 15109507

[48] Gupta M Kandula S Laxmikanth SM Vyavahare SS Reddy SBH Ramachandra CS. Controlling pain during orthodontic fixed appliance therapy with non-steroidal anti-inflammatory drugs (NSAID): a randomized, double-blinded, placebo-controlled study. J Orofac Orthop 2014;75:471–6. 10.1007/s00056-014-0243-7 25355194

[49] Hagberg C Agerberg G Hagberg M. Discomfort and bite force in painful masseter muscles after intramuscular injections of local anesthetic and saline solution. J Prosthet Dent 1986;56:354–8. 10.1016/0022-3913(86)90019-3 3462391

[50] Hagenacker T Bude V Naegel S Holle D Katsarava Z Diener HC Obermann M. Patient-conducted anodal transcranial direct current stimulation of the motor cortex alleviates pain in trigeminal neuralgia. J Headache Pain 2014;15:78. 10.1186/1129-2377-15-78 PMC4256974 25424567

[51] Hansen HJ Thorøe U. Low power laser biostimulation of chronic oro-facial pain. A double-blind placebo controlled cross-over study in 40 patients. PAIN 1990;43:169–79. 10.1016/0304-3959(90)91070-Y 1708118

[52] Harrison SD Balawi SA Feinmann C Harris M. Atypical facial pain: a double-blind placebo-controlled crossover pilot study of subcutaneous sumatriptan. Eur Neuropsychopharmacol 1997;7:83–8. 10.1016/s0924-977x(96)00385-9 9169294

[53] Hartung J Knapp G. A refined method for the meta‐analysis of controlled clinical trials with binary outcome. Stat Med 2001;20:3875–89. 10.1002/sim.1009 11782040

[54] Hochsprung A Escudero-Uribe S Ibáñez-Vera AJ Izquierdo-Ayuso G. Effectiveness of monopolar dielectric transmission of pulsed electromagnetic fields for multiple sclerosis-related pain: a pilot study. Neurologia (Engl Ed) 2021;36:433–9. 10.1016/j.nrleng.2018.03.003 34238526

[55] Hussain H Crow H Gonzalez Y McCall WD. Immediate effect of continuous ultrasound vs sham ultrasound for bilateral masseter myalgia: a double-blinded trial. J Oral Facial Pain Headache 2018;32:304–8. 10.11607/ofph.2000 29767650

[56] Ichida MC Zemuner M Hosomi J Pai HJ Teixeira MJ de Siqueira JTT de Siqueira SRDT. Acupuncture treatment for idiopathic trigeminal neuralgia: a longitudinal case-control double blinded study. Chin J Integr Med 2017;23:829–36. 10.1007/s11655-017-2786-0 29080198

[57] IntHout J Ioannidis JP Borm GF. The Hartung-Knapp-Sidik-Jonkman method for random effects meta-analysis is straightforward and considerably outperforms the standard DerSimonian-Laird method. BMC Med Res Methodol 2014;14:25. 10.1186/1471-2288-14-25 PMC4015721 24548571

[58] Ishaque M Moosa S Urban L Kundu B Qureshi Z Spears T Fletcher PT Donahue J Patel SH Goldstein RB Finan PH Liu CC Elias WJ. Bilateral focused ultrasound medial thalamotomies for trigeminal neuropathic pain: a randomized controlled study. J Neurosurg 2024;140:1799–809. 10.3171/2023.10.JNS23661 38157521

[59] Jamtøy KA Thorstensen WM Stovner LJ Rosén A Maarbjerg S Bratbak D Simpson MR Tronvik E. Onabotulinum toxin A block of the sphenopalatine ganglion in patients with persistent idiopathic facial pain: a randomized, triple-blind, placebo-controlled, exploratory, cross-over study. Cephalalgia 2023;43:3331024231187132. 10.1177/03331024231187132 37435807

[60] Jorns TP Johnston A Zakrzewska JM. Pilot study to evaluate the efficacy and tolerability of levetiracetam (Keppra) in treatment of patients with trigeminal neuralgia. Eur J Neurol 2009;16:740–4. 10.1111/j.1468-1331.2009.02585.x 19475723

[61] Kanai A Okamoto T Suzuki K Niki Y Okamoto H. Lidocaine eye drops attenuate pain associated with ophthalmic postherpetic neuralgia. Anesth Analg 2010;110:1457–60. 10.1213/ANE.0b013e3181d5adaf 20237042

[62] Kanai A Saito M Hoka S. Subcutaneous sumatriptan for refractory trigeminal neuralgia. Headache 2006;46:577–82; discussion 583–4. 10.1111/j.1526-4610.2006.00405.x 16643550

[63] Kanai A Suzuki A Osawa S Hoka S. Sumatriptan alleviates pain in patients with trigeminal neuralgia. Clin J Pain 2006;22:677–80. 10.1097/01.ajp.0000210917.18536.0d 16988562

[64] Kang SK Lee YH Park H Ro JY Auh QS. Effects of intramuscular morphine in men and women with temporomandibular disorder with myofascial pain. Oral Dis 2018;24:1591–8. 10.1111/odi.12919 PMC6193838 29920852

[65] Kaptchuk TJ Miller FG. Placebo effects in medicine. N Engl J Med 2015;373:8–9. 10.1056/NEJMp1504023 26132938

[66] Katsoulis J Ausfeld-Hafter B Windecker-Gétaz I Katsoulis K Blagojevic N Mericske-Stern R. Laser acupuncture for myofascial pain of the masticatory muscles. A controlled pilot study. Schweiz Monatsschr Zahnmed 2010;120:213–25. 20238281

[67] Keith PK Dymek A Pfaar O Fokkens W Yun Kirby S Wu W Garris C Topors N Lee LA. Fluticasone furoate nasal spray reduces symptoms of uncomplicated acute rhinosinusitis: a randomised placebo-controlled study. Prim Care Respir J 2012;21:267–75. 10.4104/pcrj.2012.00039 PMC6548020 22614920

[68] Kohútová B Fricová J Klírová M Novák T Rokyta R. Theta burst stimulation in the treatment of chronic orofacial pain: a randomized controlled trial. Physiol Res 2017;66:1041–7. 10.33549/physiolres.933474 28937248

[69] Kondziolka D Lemley T Kestle JR Lunsford LD Fromm GH Jannetta PJ. The effect of single-application topical ophthalmic anesthesia in patients with trigeminal neuralgia. A randomized double-blind placebo-controlled trial. J Neurosurg 1994;80:993–7. 10.3171/jns.1994.80.6.0993 8189280

[70] Kulekcioglu S Sivrioglu K Ozcan O Parlak M. Effectiveness of low-level laser therapy in temporomandibular disorder. Scand J Rheumatol 2003;32:114–8. 10.1080/03009740310000139 12737331

[71] Leenstra JL Miller RC Qin R Martenson JA Dornfeld KJ Bearden JD Puri DR Stella PJ Mazurczak MA Klish MD Novotny PJ Foote RL Loprinzi CL. Doxepin rinse versus placebo in the treatment of acute oral mucositis pain in patients receiving head and neck radiotherapy with or without chemotherapy: a phase III, randomized, double-blind trial (NCCTG-N09C6 [Alliance]). J Clin Oncol 2014;32:1571–7. 10.1200/JCO.2013.53.2630 PMC4026580 24733799

[72] Lefaucheur J-P Drouot X Menard-Lefaucheur I Zerah F Bendib B Cesaro P Keravel Y Nguyen J-P. Neurogenic pain relief by repetitive transcranial magnetic cortical stimulation depends on the origin and the site of pain. J Neurol Neurosurg Psychiatry 2004;75:612–6. 10.1136/jnnp.2003.022236 PMC1739005 15026508

[73] Li R Sun J Luo K Luo N Sun R Gao F Wang Y Xia Y Li X Chen L Ma R Shao X Liang Y Fang J. Electroacupuncture and carbamazepine for patients with trigeminal neuralgia: a randomized, controlled, 2 × 2 factorial trial. J Neurol 2024;271:5122–36. 10.1007/s00415-024-12433-x PMC11319385 38816482

[74] Lindholm P Lamusuo S Taiminen T Pesonen U Lahti A Virtanen A Forssell H Hietala J Hagelberg N Pertovaara A Parkkola R Jääskeläinen S. Right secondary somatosensory cortex-a promising novel target for the treatment of drug-resistant neuropathic orofacial pain with repetitive transcranial magnetic stimulation. PAIN 2015;156:1276–83. 10.1097/j.pain.0000000000000175 25830924

[75] Madani AS Ahrari F Nasiri F Abtahi M Tunér J. Low-level laser therapy for management of TMJ osteoarthritis. Cranio 2014;32:38–44. 10.1179/0886963413Z.0000000004 24660645

[76] Mansano AM Frederico TN Valentin REB Carmona MJC Ashmawi HA. Percutaneous radiofrequency ablation for trigeminal neuralgia management: a randomized, double-blinded, sham-controlled clinical trial. Pain Med 2023;24:234–43. 10.1093/pm/pnac132 36029256

[77] Martins IP Martins RP Caldas SGFR Dos Santos-Pinto A Buschang PH Pretel H. Low-level laser therapy (830 nm) on orthodontic pain: blinded randomized clinical trial. Lasers Med Sci 2019;34:281–6. 10.1007/s10103-018-2583-9 29998356

[78] Maul XA Borchard NA Hwang PH Nayak JV. Microcurrent technology for rapid relief of sinus pain: a randomized, placebo-controlled, double-blinded clinical trial. Int Forum Allergy Rhinol 2019;9:352–6. 10.1002/alr.22280 PMC6590214 30667597

[79] Mazzetto MO Hotta TH Pizzo RCDA. Measurements of jaw movements and TMJ pain intensity in patients treated with GaAlAs laser. Braz Dent J 2010;21:356–60. 10.1590/s0103-64402010000400012 20976388

[80] Meltzer EO Gates D Bachert C. Mometasone furoate nasal spray increases the number of minimal-symptom days in patients with acute rhinosinusitis. Ann Allergy Asthma Immunol 2012;108:275–9. 10.1016/j.anai.2012.01.015 22469449

[81] Menhinick KA Gutmann JL Regan JD Taylor SE Buschang PH. The efficacy of pain control following nonsurgical root canal treatment using ibuprofen or a combination of ibuprofen and acetaminophen in a randomized, double-blind, placebo-controlled study. Int Endod J 2004;37:531–41. 10.1111/j.1365-2591.2004.00836.x 15230906

[82] Misra P Nayak C Chattopadhyay A Palit TK Gupta B Sadhukhan S Bhar K Rai S Parewa M Ali SS Basu A Nath A Koley M Saha S. Individualized homeopathic medicines in chronic rhinosinusitis: randomized, double-blind, placebo-controlled trial. Homeopathy 2021;110:13–26. 10.1055/s-0040-1715842 32869214

[83] Monteiro L Ferreira R Resende T Pacheco JJ Salazar F. Effectiveness of photobiomodulation in temporomandibular disorder-related pain using a 635 nm diode laser: a randomized, blinded, and placebo-controlled clinical trial. Photobiomodul Photomed Laser Surg 2020;38:280–8. 10.1089/photob.2019.4730 32427553

[84] Montonen M Kalso E Pylkkären L Lindströrm BM Lindqvist C. Disodium clodronate in the treatment of diffuse sclerosing osteomyelitis (DSO) of the mandible. Int J Oral Maxillofac Surg 2001;30:313–7. 10.1054/ijom.2001.0061 11518354

[85] Moreno ML Percival SS Kelly DL Dahl WJ. Daily olive oil intake is feasible to reduce trigeminal neuralgia facial pain: a pilot study. Nutr Res 2024;123:101–10. 10.1016/j.nutres.2024.01.005 38306883

[86] Niki Y Kanai A Hoshi K Okamoto H. Immediate analgesic effect of 8% lidocaine applied to the oral mucosa in patients with trigeminal neuralgia. Pain Med 2014;15:826–31. 10.1111/pme.12349 24506194

[87] Pfaar O Mullol J Anders C Hörmann K Klimek L. Cyclamen europaeum nasal spray, a novel phytotherapeutic product for the management of acute rhinosinusitis: a randomized double-blind, placebo-controlled trial. Rhinology 2012;50:37–44. 10.4193/Rhino11.141 22469604

[88] Planas ME Gay-Escoda C Bagán JV Santamaría J Peñarrocha M Donado M Puerta JL García-Magaz I Ruíz J Ortiz P. Oral metamizol (1 g and 2 g) versus ibuprofen and placebo in the treatment of lower third molar surgery pain: randomised double-blind multi-centre study. Cooperative Study Group. Eur J Clin Pharmacol 1998;53:405–9. 10.1007/s002280050400 9580448

[89] Posit team. RStudio: integrated development environment for R. Boston, MA, USA: Posit Software, PBC; http://www.posit.co/ 2025, Accessed August 26, 2026.

[90] Pramod GV Shambulingappa P Shashikanth MC Lele S. Analgesic efficacy of diazepam and placebo in patients with temporomandibular disorders: a double blind randomized clinical trial. Indian J Dent Res 2011;22:404–9. 10.4103/0970-9290.87062 22048580

[91] Rampello A Papi P Pompa G Rampello A Polimeni A Di Paolo C. A novel universal device “LINGUAL RING Ri.P.A.Ra” for TMDs and cranio-cervico-mandibular pains: preliminary results of a randomized control clinical trial. Eur Rev Med Pharmacol Sci 2018;22:1180–90. 10.26355/eurrev_201803_14456 29565472

[92] Reeve GS Insel O Thomas C Houle AN Miloro M. Does the use of botulinum toxin in treatment of myofascial pain disorder of the masseters and temporalis muscles reduce pain, improve function, or enhance quality of life? J Oral Maxillofac Surg 2024;82:393–401. 10.1016/j.joms.2023.12.014 PMC11648603 38228287

[93] Reid KI Dionne RA Sicard-Rosenbaum L Lord D Dubner RA. Evaluation of iontophoretically applied dexamethasone for painful pathologic temporomandibular joints. Oral Surg Oral Med Oral Pathol 1994;77:605–9. 10.1016/0030-4220(94)90319-0 8065724

[94] Rezazadeh F Borhani-Haghighi A Mosallanejad F Showraki N Tabesh A Khorami ET. Effect of non-ablative non-thermal CO2 laser on drug-resistant trigeminal neuralgia: a pilot study. Photobiomodul Photomed Laser Surg 2023;41:272–6. 10.1089/photob.2023.0023 37335618

[95] Rizzatti-Barbosa CM Nogueira MTP de Andrade ED Ambrosano GMB de Barbosa JRA. Clinical evaluation of amitriptyline for the control of chronic pain caused by temporomandibular joint disorders. Cranio 2003;21:221–5. 10.1080/08869634.2003.11746254 12889679

[96] Roth PM Thrash WJ. Effect of transcutaneous electrical nerve stimulation for controlling pain associated with orthodontic tooth movement. Am J Orthod Dentofacial Orthop 1986;90:132–8. 10.1016/0889-5406(86)90045-4 3488674

[97] Santana-Penín U Santana-Mora U López-Solache A Mora MJ Collier T Pocock SJ Lorenzo-Franco F Varela-Centelles P López-Cedrún JL. Remodeling dental anatomy vs sham therapy for chronic temporomandibular disorders. A placebo-controlled randomized clinical trial. Ann Anat 2023;250:152117. 10.1016/j.aanat.2023.152117 37302432

[98] Schott Andersen AS Maarbjerg S Noory N Heinskou TB Forman JL Cruccu G Ashina M Bendtsen L. Safety and efficacy of erenumab in patients with trigeminal neuralgia in Denmark: a double-blind, randomised, placebo-controlled, proof-of-concept study. Lancet Neurol 2022;21:994–1003. 10.1016/S1474-4422(22)00294-0 36113495

[99] Schwartz G Galonski M Gordon A Shandling M Mock D Tenenbaum HC. Effects of salmon calcitonin on patients with atypical (idiopathic) facial pain: a randomized controlled trial. J Orofac Pain 1996;10:306–15. 9161235

[100] Scrivani S Wallin D Moulton EA Cole S Wasan AD Lockerman L Bajwa Z Upadhyay J Becerra L Borsook D. A fMRI evaluation of lamotrigine for the treatment of trigeminal neuropathic pain: pilot study. Pain Med 2010;11:920–41. 10.1111/j.1526-4637.2010.00859.x 20492571

[101] Shehata HS El-Tamawy MS Shalaby NM Ramzy G. Botulinum toxin-type A: could it be an effective treatment option in intractable trigeminal neuralgia? J Headache Pain 2013;14:92. 10.1186/1129-2377-14-92 PMC4177416 24251833

[102] Shimada A Castrillon EE Svensson P. Revisited relationships between probable sleep bruxism and clinical muscle symptoms. J Dent 2019;82:85–90. 10.1016/j.jdent.2019.01.013 30716450

[103] Silver M Blum D Grainger J Hammer AE Quessy S. Double-blind, placebo-controlled trial of lamotrigine in combination with other medications for neuropathic pain. J Pain Symptom Manage 2007;34:446–54. 10.1016/j.jpainsymman.2006.12.015 17662571

[104] Simma I Gleditsch JM Simma L Piehslinger E. Immediate effects of microsystem acupuncture in patients with oromyofacial pain and craniomandibular disorders (CMD): a double-blind, placebo-controlled trial. Br Dent J 2009;207:E26. 10.1038/sj.bdj.2009.959 19876045

[105] Simpson DM Olney R McArthur JC Khan A Godbold J Ebel-Frommer K. A placebo-controlled trial of lamotrigine for painful HIV-associated neuropathy. Neurology 2000;54:2115–9. 10.1212/wnl.54.11.2115 10851374

[106] Singer E Dionne R. A controlled evaluation of ibuprofen and diazepam for chronic orofacial muscle pain. J Orofac Pain 1997;11:139–46. 10332320

[107] Sipahi A Satilmis T Basa S. Comparative study in patients with symptomatic internal derangements of the temporomandibular joint: analgesic outcomes of arthrocentesis with or without intra-articular morphine and tramadol. Br J Oral Maxillofac Surg 2015;53:316–20. 10.1016/j.bjoms.2014.12.018 25623934

[108] Smith P Mosscrop D Davies S Sloan P Al-Ani Z. The efficacy of acupuncture in the treatment of temporomandibular joint myofascial pain: a randomised controlled trial. J Dent 2007;35:259–67. 10.1016/j.jdent.2006.09.004 17095133

[109] Sohrabi C Franchi T Mathew G Kerwan A Nicola M Griffin M Agha M Agha R. PRISMA 2020 statement: what's new and the importance of reporting guidelines. Int J Surg 2021;88:105918. 10.1016/j.ijsu.2021.105918 33789825

[110] Sperber SJ Turner RB Sorrentino JV O'Connor RR Rogers J Gwaltney JM. Effectiveness of pseudoephedrine plus acetaminophen for treatment of symptoms attributed to the paranasal sinuses associated with the common cold. Arch Fam Med 2000;9:979–85. 10.1001/archfami.9.10.979 11115196

[111] Starling AJ Cowan RP Buse DC Diener H-C Marmura MJ Hirman J Brevig T Cady R. Eptinezumab improved patient-reported outcomes in patients with migraine and medication-overuse headache: subgroup analysis of the randomized PROMISE-2 trial. Headache 2023;63:264–74. 10.1111/head.14434 36633219

[112] Stavropoulou E Argyra E Zis P Vadalouca A Siafaka I. The effect of intravenous lidocaine on trigeminal neuralgia: a randomized double blind placebo controlled trial. ISRN Pain 2014;2014:853826. 10.1155/2014/853826 PMC4893393 27335883

[113] Storari M Zerman N Salis B Spinas E. Chronic facial pain in fibromyalgia: may electromagnetic field represent a promising new therapy? A pilot randomized-controlled study. Int J Environ Res Public Health 2022;20:391. 10.3390/ijerph20010391 PMC9819752 36612711

[114] Ta LE Dionne RA. Treatment of painful temporomandibular joints with a cyclooxygenase-2 inhibitor: a randomized placebo-controlled comparison of celecoxib to naproxen. PAIN 2004;111:13–21. 10.1016/j.pain.2004.04.029 15327804

[115] Tchivileva IE Ohrbach R Fillingim RB Lim PF Giosia MD Ribeiro-Dasilva M Campbell JH Hadgraft H Willis J Arbes SJ Slade GD. Effect of comorbid migraine on propranolol efficacy for painful TMD in a randomized controlled trial. Cephalalgia 2021;41:839–50. 10.1177/0333102421989268 PMC8166404 33560875

[116] Tepper SJ Grosberg B Daniel O Kuruvilla DE Vainstein G Deutsch L Sharon R. Migraine treatment with external concurrent occipital and trigeminal neurostimulation-A randomized controlled trial. Headache 2022;62:989–1001. 10.1111/head.14350 35748757

[117] Tjakkes GHE Tenvergert EM de Bont LGM Stegenga B. The effect of intra-articular injection of ultracain in the temporomandibular joint in patients with preauricular pain: a randomized prospective double-blind placebo-controlled crossover study. Clin J Pain 2007;23:233–6. 10.1097/AJP.0b013e31802f0950 17314582

[118] Tuttle AH Tohyama S Ramsay T Kimmelman J Schweinhardt P Bennett GJ Mogil JS. Increasing placebo responses over time in U.S. clinical trials of neuropathic pain. PAIN 2015;156:2616–26. 10.1097/j.pain.0000000000000333 26307858

[119] Varoni EM Lo Faro AF Lodi G Carrassi A Iriti M Sardella A. Melatonin treatment in patients with burning mouth syndrome: a triple-blind, placebo-controlled, crossover randomized clinical trial. J Oral Facial Pain Headache 2018;32:178–88. 10.11607/ofph.1913 29694465

[120] Venekamp RP Bonten MJM Rovers MM Verheij TJM Sachs APE. Systemic corticosteroid monotherapy for clinically diagnosed acute rhinosinusitis: a randomized controlled trial. CMAJ 2012;184:E751–7. 10.1503/cmaj.120430 PMC3470641 22872770

[121] Venezian GC da Silva MAMR Mazzetto RG Mazzetto MO. Low level laser effects on pain to palpation and electromyographic activity in TMD patients: a double-blind, randomized, placebo-controlled study. Cranio 2010;28:84–91. 10.1179/crn.2010.012 20491229

[122] Viechtbauer W. Bias and efficiency of meta-analytic variance estimators in the random-effects model. J Educ Behav Stat 2005;30:261–93.

[123] von Lindern JJ Niederhagen B Bergé S Appel T. Type A botulinum toxin in the treatment of chronic facial pain associated with masticatory hyperactivity. J Oral Maxillofac Surg 2003;61:774–8. 10.1016/s0278-2391(03)00153-8 12856249

[124] Wager TD Atlas LY. The neuroscience of placebo effects: connecting context, learning and health. Nat Rev Neurosci 2015;16:403–18. 10.1038/nrn3976 PMC6013051 26087681

[125] Wan C Dong DS Song T. High-voltage, long-duration pulsed radiofrequency on gasserian ganglion improves acute/subacute zoster-related trigeminal neuralgia: a randomized, double-blinded, controlled trial. Pain Physician 2019;22:361–8. 31337167

[126] Wartolowska K Judge A Hopewell S Collins GS Dean BJF Rombach I Brindley D Savulescu J Beard DJ Carr AJ. Use of placebo controls in the evaluation of surgery: systematic review. BMJ 2014;348:g3253. 10.1136/bmj.g3253 PMC4029190 24850821

[127] Wu CJ Lian YJ Zheng YK Zhang HF Chen Y Xie NC Wang LJ. Botulinum toxin type A for the treatment of trigeminal neuralgia: results from a randomized, double-blind, placebo-controlled trial. Cephalalgia 2012;32:443–50. 10.1177/0333102412441721 22492424

[128] Zakrzewska JM Chaudhry Z Nurmikko TJ Patton DW Mullens LE. Lamotrigine (lamictal) in refractory trigeminal neuralgia: results from a double-blind placebo controlled crossover trial. PAIN 1997;73:223–30. 10.1016/S0304-3959(97)00104-8 9415509

[129] Zakrzewska JM Palmer J Morisset V Giblin GM Obermann M Ettlin DA Cruccu G Bendtsen L Estacion M Derjean D Waxman SG Layton G Gunn K Tate S, study investigators. Safety and efficacy of a Nav1.7 selective sodium channel blocker in patients with trigeminal neuralgia: a double-blind, placebo-controlled, randomised withdrawal phase 2a trial. Lancet Neurol 2017;16:291–300. 10.1016/S1474-4422(17)30005-4 28216232

[130] Zhang H Lian Y Ma Y Chen Y He C Xie N Wu C. Two doses of botulinum toxin type A for the treatment of trigeminal neuralgia: observation of therapeutic effect from a randomized, double-blind, placebo-controlled trial. J Headache Pain 2014;15:65. 10.1186/1129-2377-15-65 PMC4194456 25263254

[131] Zhang Y Zhang J Wang L Wang K Svensson P. Effect of transcutaneous electrical nerve stimulation on jaw movement-evoked pain in patients with TMJ disc displacement without reduction and healthy controls. Acta Odontol Scand 2020;78:309–20. 10.1080/00016357.2019.1707868 31876451

[132] Zhang YP Wang Y Xia WG Song AQ. Triple puncture for primary trigeminal neuralgia: a randomized clinical trial. Curr Med Sci 2019;39:638–44. 10.1007/s11596-019-2085-4 31347002

[133] Zhao D Li J Ma C Huang Y Tao G. Cardiovascular response to Gasserian ganglion ablation on trigeminal neuralgia under local anesthesia: a retrospective single-blind case-control study. BMC Anesthesiol 2022;22:104. 10.1186/s12871-022-01644-2 PMC8996585 35410169

[134] Ziegler CM Wiechnik J Mühling J. Analgesic effects of intra-articular morphine in patients with temporomandibular joint disorders: a prospective, double-blind, placebo-controlled clinical trial. J Oral Maxillofac Surg 2010;68:622–7. 10.1016/j.joms.2009.04.049 20171481

[135] Zotelli VL Grillo CM Gil ML Wada RS Sato JE da Luz Rosário de Sousa M. Acupuncture effect on pain, mouth opening limitation and on the energy meridians in patients with temporomandibular dysfunction: a randomized controlled trial. J Acupunct Meridian Stud 2017;10:351–9. 10.1016/j.jams.2017.08.005 29078971

[136] Zúñiga C Piedimonte F Díaz S Micheli F. Acute treatment of trigeminal neuralgia with onabotulinum toxin A. Clin Neuropharmacol 2013;36:146–50. 10.1097/WNF.0b013e31829cb60e 24045604

