# Supplementary material for: A meta-analysis of pain intensity changes after placebo interventions in facial pain
Source: Pain Rep. 2026 Oct 5;11(6):e1499. doi: 10.1097/PR9.0000000000001499 (PMC13641656; doi:10.1097/PR9.0000000000001499)
Supplement: Supplementary file 1 [file painreports-11-e1499-s001.pdf]

Supplementary File

Supplemental Table 1 - Meta-analysis primary study characteristics

Study	Age (mean)	% Female	Pain type	Intervention type	Intervention (specific)	Patients (N)	Effect Size	CI Lower	CI Upper	Pain duration, years (Mean, SD)	Follow-up (months)
**Ishaque et al., 2024**	67.40	80.00	Trigeminal Neuropathic Pain	Surgery	FUS medial thalamotomy	10	10.26	9.45	11.06	14.20 (10.30)	3.00
**Jamtøy et al., 2023**	51.60	17.00	Atypical Facial Pain	Injection or nerve block	BTA injection	29	9.00	2.90	15.10	NS	5.00
**Tepper et al., 2022**	40.30	83.00	TMD	Surgery	eCOT-NS (external concurrent occipital and trigeminal neurostimulation)	59	36.84	21.05	52.63	NS	NS
**Tchivileva et al., 2020**	34.05	77.50	TMD	Medication	Propranolol	87	52.6	41.4	63.8	10.50 (9.43)	0.23
**Tchivileva et al., 2021**	34.40	74.70	TMD	Medication	Propranolol	46	10.4	6.1	14.7	10.20 (8.80)	0.23
**Harrison et al., 1997**	18-65	NS	Atypical Facial Pain	Medication	Sumatriptan	17	-2.66	-8.4	3.08	NS	NS
**Zakrzewska et al., 2017**	54.50	72.00	Classical Trigeminal Neuralgia	Medication	BIIB074 (sodium-channel blocker)	29	46.58	20.84	72.33	7(1-11) Median (IQR)	0.23
**Misra et al., 2020**	33.70	51.60	TMD	Alternative	Homeopathy	31	52.00	23.68	80.32	8.1 (6.8)	16.00
**Venekamp et al., 2012**	43.15	61.60	TMD	Medication	Prednisolone	92	55.80	55.51	56.09	0.04 (0.02-0.06) Median (IQR)	0.47
**Silver et al., 2007**	60.25	45.00	Classical Trigeminal Neuralgia	Medication	Lamotrigine	106	33.33	26.69	39.98	4.9 (5.4)	3.50
**Leenstra et al., 2014**	61.00	20.00	TMD	Medication	Doxepin	78	17.54	17.19	17.90	NS	NS
**Starling et al., 2023**	41.40	87.00	TMD	Medication	Eptinezumab	145	6.27	-9.87	22.40	NS	24.00
**Hussain et al., 2018**	37.65	100.00	TMD	Alternative	Ultrasound	10	17.02	-22.19	56.23	NS	NS
**Kondziolka et al., 1994**	59.00	70.20	Classical Trigeminal Neuralgia	Medication	Ophthalmic anesthetic eye drops	22	51.36	41.40	61.31	NS	1.00
**Keith et al., 2012**	39.30	62.00	TMD	Medication	FFNS (fluticasone furoate nasal spray)	245	58.17	58.15	58.18	NS	2.00
**Gremeau-Richard et al., 2004**	65.40	91.60	TMD	Medication	Clonazepam	24	9.68	7.10	12.26	NS	0.47
**Sperber et al., 2000**	28.10	66.27	TMD	Medication	Pseudoephedrine and Acetaminophen	214	9.5	7.89	11.11	NS	0.00
**Niki et al., 2014**	69.00	75.00	Classical Trigeminal Neuralgia	Injection or nerve block	Lidocaine	24	0.00	-6.00	6.00	8.7 (4-15.8) Median (IQR)	NS

**Schwartz et al., 1996**	47.38	100.00	Atypical Facial Pain	Alternative	Salmon Calcitonin	13	0.40	-0.34	1.14	NS	2.00
**Mansano et al., 2022**	64.70	73.35	Classical Trigeminal Neuralgia	Surgery	Radiofrequency	15	79	74.40	83.60	1.15 (1.02-1.35) Median (IQR)	12.00
**Ebrahimi et al., 2018**	NS	63.33	Classical Trigeminal Neuralgia	Alternative	LLLT (low-level laser therapy)	15	34	18.70	49.3	NS	4.00
**Hagenacker et al., 2014**	63.00	50.00	Classical Trigeminal Neuralgia	Noninvasive electrical stimulation	tDCS (transcranial direct current stimulation)	10	-8.33	-26.96	10.29	13.0 (8.9)	0.47
**Schott Andersen et al., 2022**	56.45	71.50	Classical Trigeminal Neuralgia	Medication	Erenumab	40	51.36	41.40	61.31	7 (3-11) Median (IQR)	1.00
**Reeve et al., 2024**	36.00	81.00	TMD	Injection or nerve block	BTA injection	37	33.33	32.62	34.04	NS	3.00
**Wu et al., 2012**	58.60	54.76	Classical Trigeminal Neuralgia	Injection or nerve block	BTA injection	19	24.42	23.41	25.43	5.83 (6.67)	3.00
**Gazerani et al., 2008**	26.30	0.00	Trigeminal Neuropathic Pain	Injection or nerve block	BTA injection	8	1.43	-18.71	21.57	NS	0.23
**Forssell et al., 2004**	52.00	60.00	Atypical Facial Pain	Medication	Venlafaxine	18	-4.44	-35.67	26.78	NS	NS
**Madani et al., 2014**	47.50	95.00	TMD	Noninvasive electrical stimulation	LLLT (low-level laser therapy)	10	36.55	10.62	62.47	NS	1.00
**Scrivani et al., 2010**	48.30	66.60	Trigeminal Neuropathic Pain	Medication	Lamotrigine	6	14.06	-26.51	54.63	NS	NS
**Monteiro et al., 2020**	27.40	32.00	TMD	Noninvasive electrical stimulation	Photobiomodulation	20	8.99	-25.79	43.77	0.55 (0.38)	1.00
**Dolwick et al., 2020**	48.90	100.00	TMD	Medication	triamcinolone acetonide	10	48.35	47.17	49.52	NS	3.00
**Emshoff et al., 2008**	42.95	75.00	TMD	Noninvasive electrical stimulation	LLLT (low-level laser therapy)	26	70.28	50.13	90.42	0.51 (0.20)	2.00
**Maul et al., 2019**	44.10	63.40	Atypical Facial Pain	Noninvasive electrical stimulation	active microcurrent-treated	33	15.00	3.28	26.72	NS	NS
**Simma et al., 2009**	41.00	100.00	TMD	Alternative	Acupuncture	12	26.83	-18.23	71.89	NS	NS
**Martins et al., 2018**	19.80	58.00	TMD	Noninvasive electrical stimulation	LLLT (low-level laser therapy)	31	31.25	-13.89	76.39	NS	0.23
**Zhang et al., 2014**	59.81	55.00	Classical Trigeminal Neuralgia	Injection or nerve block	BTA injection	28	49.71	48.98	50.44	4.25 (3.86)	2.00
**Kang et al., 2018**	28.50	47.00	TMD	Medication	Morphine	11	26.19	-15.60	67.98	NS	NS
**Arantes et al., 2009**	20.50	not specified	TMD	Medication	Tenoxicam	12	56.28	13.17	99.39	NS	1.00
**De Icco et al., 2020**	48.30	80.00	TMD	Noninvasive electrical stimulation	tDCS (transcranial direct current stimulation)	10	14.63	-49.54	78.81	7.6 (7.1)	1.00
**Kohútová et al., 2017**	57.30	63.00	TMD	Noninvasive electrical stimulation	Theta Burst Stimulation (form of rTMS)	9	3.33	-20.22	26.89	NS	0.50

**Zotelli et al., 2017**	36.50	80.00	TMD	Alternative	Acupuncture	20	76.32	51.99	100.65	7.6 (7.4)	NS
**Katsoulis et al., 2010**	33.00	NS	TMD	Alternative	Laser Acupuncture	4	45.64	15.56	75.73	NS	3.00
**Venezian et al., 2010**	41.58	89.60	TMD	Noninvasive electrical stimulation	LLLT (low-level laser therapy)	24	11.70	4.98	18.42	NS	1.00
**Dias et al., 2022**	33.42	79.40	TMD	Noninvasive electrical stimulation	photobiomodulation combined with orofacial myofunctional therapy	15	46.60	22.50	70.70	NS	3.00
**Wan et al., 2019**	65.44	55.20	Classical Trigeminal Neuralgia	Surgery	PRF (pulsed radiofrequency)	47	34.34	33.65	35.02	NS	6.00
**Simpson et al., 2000**	44.50	15.55	Trigeminal Neuropathic Pain	Medication	Lamotrigine	20	16.19	-7.10	39.48	NS	3.50
**Singer and Dionne, 1997**	36.10	89.70	TMD	Medication	Diazepam and ibuprofen	10	40.05	-29.08	109.18	NS	1.00
**Varoni et al., 2018**	64.40	80.00	Trigeminal Neuropathic Pain	Medication	Melatonin	20	14.10	11.86	16.35	NS	NS
**Fricová et al., 2013**	50.70	69.50	Atypical Facial Pain	Noninvasive electrical stimulation	rTMS (repetitive transcranial magnetic stimulation)	10	-3.45	-23.04	16.15	NS	0.47
**Tjakkes et al., 2007**	32.50	95.00	TMD	Injection or nerve block	Ultracain	19	-7.79	-37.16	21.59	NS	NS
**Pramod et al., 2011**	NS	60.00	TMD	Medication	Diazepam	10	67.15	41.33	92.98	NS	NS
**Montonen et al., 2001**	49.60	80.00	TMD	Medication	Disodium Clodronate	4	27.30	5.00	49.50	8.5 (5.83)	12.00
**Chiu and Cheung, 2005**	22.64	70.90	TMD	Medication	Ibuprofen	32	24.76	24.07	25.44	NS	NS
**Ziegler et al., 2010**	50.00	NS	TMD	Medication	Morphine	24	34.72	13.17	56.28	NS	24.00
**Ta and Dionne, 2004**	34.26	67.66	TMD	Medication	Naproxen	22	34.86	24.65	45.07	4.0 (4.7)	2.00
**Amanat et al., 2013**	47.22	73.33	TMD	Noninvasive electrical stimulation	GaAs laser	23	49.33	29.59	69.08	NS	4.00
**Sipahi et al., 2015**	33.00	83.33	TMD	Medication	Morphine	10	62.32	36.36	88.28	NS	6.00
**Pfaar et al., 2012**	39.65	64.60	TMD	Medication	Cyclamen europaeum (nasal spray)	50	47.94	26.34	69.46	NS	0.50
**Smith et al., 2006**	40.50	51.85	TMD	Alternative	Acupuncture	12	0.83	-2.50	4.20	4.4 (2.8)	NS
**Ichida et al., 2017**	51.50	NS	Classical Trigeminal Neuralgia	Alternative	Acupuncture	15	20.69	-54.67	96.05	NS	6.00
**Stavropoulou et al., 2014**	65.20	65.00	Classical Trigeminal Neuralgia	Medication	Lidocaine	40	38.90	22.57	55.22	NS	0.03
**Cahlin and Dahlström, 2011**	57.25	86.40	TMD	Medication	Glucosamine sulfate	29	13.41	-16.32	43.14	NS	NS

**Bittar and Graff-Radford, 1993**	48.70	65.00	Classical Trigeminal Neuralgia	Injection or nerve block	Lidocaine	20	52.84	33.96	71.73	NS	30.00
**Zúñiga et al., 2013**	65.28	47.22	Classical Trigeminal Neuralgia	Injection or nerve block	BTA injection	16	15.26	-3.62	34.15	5.2 (3.1)	1.00
**Bisla et al., 2021**	55.50	51.92	Classical Trigeminal Neuralgia	Noninvasive electrical stimulation	TENS (transcutaneous electrical nerve stimulation)	26	76.13	69.97	82.28	NS	12.00
**Gupta et al., 2014**	not given; ages 15-22	51.10	TMD	Medication	Acetaminophen	15	-15.91	-32.50	0.68	NS	NS
**Li et al., 2024**	58.50	62.50	Classical Trigeminal Neuralgia	Noninvasive electrical stimulation	Electroacupuncture	29	26.67	19.57	33.77	60.0 (14.9)	7.00
**Bertolami et al., 1993**	38.35	88.42	TMD	Medication	Sodium Hyaluronate	60	37.78	16.22	59.34	7.9	6.00
**Menhinick et al., 2004**	41.00	64.10	TMD	Medication	Ibuprofen	19	60.00	58.25	61.75	NS	NS
**Gerschman et al., 1994**	NS	NS	TMD	Noninvasive electrical stimulation	LLLT (low-level laser therapy)	28	21.15	-0.38	42.69	NS	NS
**Planas et al., 1998**	not given; ages 18-60	NS	TMD	Medication	Ibuprofen	32	45.56	28.59	62.54	NS	NS

Supplemental Table 2 – Uncontrolled case studies characteristics

**Study**	**Age (mean)**	**% Female**	**Pain type**	**Intervention type**	**Intervention (specific)**	**Patients (N)**	**Effect Size**	**CI Lower**	**CI Upper**	**Follow-up (months)**
**Santana-Penín et al., 2023**	29.50	35.50	TMD	Alternative	ET	38	62.86	50.59	75.12	6.00
**Feinmann et al., 1984**	40.50	78.50	Atypical Facial Pain	Medication	Dothiepin	45	36.36	27.07	45.66	12.00
**Diener et al., 2021**	41.40	87.20	TMD	Medication	Eptinezumab	145	34.73	27.82	41.64	24.00
**Shimada et al., 2019**	18.00	NS	TMD	Noninvasive electrical stimulation	CES (contingent electrical stimulation)	20	0	-7.91	-7.47	0.47
**Zakrzewska et al., 1998**	60.00	42.90	Classical Trigeminal Neuralgia	Medication	LTG (lamotrigine)	14	47.06	46.01	48.11	NS

**Agius et al., 2013**	35.66	74.00	Atypical Facial Pain	Medication	amitriptyline	20	30.34	7.92	52.77	36.00
**Antonio et al., 2011**	27.70	NS	TMD	Medication	Citrate	18	0.00	-80.24	80.24	NS
**Zhang et al., 2019**	32.50	50.00	TMD	Noninvasive electrical stimulation	TENS (transcutaneous electrical nerve stimulation)	20	-9.09	-12.06	-6.12	3.00
**DeVocht et al., 2013**	32.40	79.00	TMD	Alternative	AMCT (activator method chiropractic technique)	20	45.71	20.00	71.43	6.00
**Lindholm et al., 2015**	59.00	90.00	Trigeminal Neuropathic Pain	Noninvasive electrical stimulation	rTMS (repetitive transcranial magnetic stimulation)	20	-3.09	-3.27	-2.92	1.00
**Hagberg et al., 1986**	27.00	100.00	TMD	Injection or nerve block	Lidocaine	30	56.00	42.00	70.00	0.23
**Rampello et al., 2018**	36.00	86.25	TMD	Alternative	Lingual Ring device	40	-8.00	-28.14	12.14	3.00
**al Balawi et al., 1996**	41.50	84.21	Atypical Facial Pain	Medication	Sumatriptan	19	17.60	14.53	20.66	NS
**Moreno et al., 2024**	53.00	100%	Classical Trigeminal Neuralgia	Alternative	Extra virgin olive oil	24	8.89	2.60	15.18	4.00
**Zhang et al., 2019**	46.20	64.00	Classical Trigeminal Neuralgia	Alternative	Acupuncture	30	48.00	37.26	58.74	3.00
**Storari et al., 2023**	54.00	100.00	Atypical Facial Pain	Noninvasive electrical stimulation	EMF (Electromagnetic frequency)	9	5.26	3.96	6.57	12.00
**Zhao et al., 2022**	67.35	51.00	Classical Trigeminal Neuralgia	Surgery	RFT (radiofrequency thermocoagulation)	40	57.14	56.36	57.93	NS
**Kanai et al., 2006**	64.10	86.60	Classical Trigeminal Neuralgia	Medication	Oral sumatriptan	15	35.00	18.00	52.00	0.47
**Goddard et al., 2002**	35.01	83.75	Atypical Facial Pain	Alternative	Acupuncture	8	29.08	4.62	53.53	NS
**Gao et al., 2019**	63.97	38.50	Classical Trigeminal Neuralgia	Alternative	Acupuncture	54	2.44	-30.69	35.57	2.00
**Rezazadeh et al., 2023**	51.70	50.00	Classical Trigeminal Neuralgia	Alternative	NANTCL (non-ablative non-thermal CO2 laser)	12	7.82	-11.37	27.02	3.00
**Barbosa et al., 2019**	28.00	100.00	TMD	Alternative	Biting endurance exercises measured by surface electromyography	23	38.78	3.08	74.47	2.00
**Demirkol et al., 2014**	NS	NS	TMD	Noninvasive electrical stimulation	LLLT (low-level laser therapy)	10	10.81	-17.64	39.26	1.00
**Kanai et al., 2006**	63.00	83.00	Classical Trigeminal Neuralgia	Medication	Sumatriptan	NS	4.71	-31.99	41.40	NS

**Alencar et al., 2014**	36.83	95.00	TMD	Medication	Cyclobenzaprine/tizanidine	15	27.69	13.17	42.22	1.00
**da Cunha et al., 2008**	43.38	97.50	TMD	Noninvasive electrical stimulation	LLLT (low-level laser therapy)	20	29.24	8.07	50.41	1.00
**Kulekcioglu et al., 2003**	38.10	80.00	TMD	Noninvasive electrical stimulation	LLLT (low-level laser therapy)	15	77.34	68.83	85.85	1.00
**Lefaucheur et al., 2004**	54.60	53.30	Trigeminal Neuropathic Pain	Noninvasive electrical stimulation	rTMS	60	8.82	7.48	10.17	NS
**Ferreira et al., 2017**	24.63	75.00	TMD	Noninvasive electrical stimulation	TENS (transcutaneous electrical nerve stimulation)	20	14.29	-56.56	85.13	2.50
**Reid et al., 1994**	39.55	81.10	TMD	Medication	dexamethasone with lidocaine	31	24.44	13.17	35.72	0.60
**Rizzatti-Barbosa et al., 2003**	NS	100.00	TMD	Medication	Amitriptyline	6	34.72	13.17	56.28	NS
**Eslamian et al., 2013**	23.00	40.00	TMD	Medication	Benzocaine patches	30	50.88	50.55	51.21	NS
**Curtis et al., 1994**	29.70	NS	TMD	Injection or nerve block	Ketorolac Tromethamine	20	5.06	4.77	5.34	NS
**Hansen and Thorøe, 1990**	59.00	92.50	Atypical Facial Pain	Noninvasive electrical stimulation	IR diode laser	40	79.58	60.40	98.77	6.00
**Mazzetto et al., 2010**	NS	NS	TMD	Noninvasive electrical stimulation	LLLT (low-level laser therapy)	20	1.16	-5.87	8.19	NS
**Jorns et al., 2009**	58.20	40.00	Classical Trigeminal Neuralgia	Medication	Levetiracetam	5	11.69	-18.02	41.40	2.50
**Kanai et al., 2010**	73.00	54.16	Classical Trigeminal Neuralgia	Injection or nerve block	Topical Lidocaine	12	17.24	-16.57	51.05	0.23
**Hochsprung et al., 2021**	46.75	50.00	TMD	Noninvasive electrical stimulation	PEMF (pulsed electromagnetic fields)	12	0.18	-34.42	34.78	1.00
**Cetiner et al., 2006**	33.80	91.70	TMD	Noninvasive electrical stimulation	LLLT (low-level laser therapy)	15	21.00	-0.35	42.36	1.00
**Arikan et al., 2005**	30.90	45.00	TMD	Alternative	Cryotherapy	10	64.58	12.15	52.43	NS
**DeNucci et al., 1998**	39.20	90.00	TMD	Medication	Triazolam	10	16.67	-13.26	46.59	0.47
**Roth and Thrash, 1986**	28.00	40.00	TMD	Noninvasive electrical stimulation	TENS (transcutaneous electrical nerve stimulation)	18	40.80	-5.87	87.47	0.13
**Da Silva et al., 2012**	39.70	66.60	TMD	Noninvasive electrical stimulation	LLLT (low-level laser therapy)	15	-11.20	-31.08	8.69	1.50
**Shehata et al., 2013**	45.95	55.00	Classical Trigeminal Neuralgia	Injection or nerve block	BTA injection	20	3.53	-26.89	33.96	3.00

Supplemental Table 3– Characteristics of Placebo Procedures in Surgical and Interventional Studies

Study	Intervention type	Intervention (specific)	Description of the Placebo Intervention
**Ishaque et al., 2024**	Surgery	FUS medial thalamotomy	Participants underwent identical procedural workflow, including stereotactic head-frame placement, MRI-guided targeting, transducer positioning, and intraoperative assessments. Acoustic power was disabled, and no therapeutic ultrasound energy was delivered.
**Jamtøy et al., 2023**	Injection or nerve block	BTA injection	Navigation-assisted injection of 0.5 mL isotonic saline toward the sphenopalatine ganglion (SPG) on the affected side using the same MultiGuide® device, injection approach, syringe volume, and procedural protocol as the active treatment. An independent nurse prepared identical syringes to maintain triple blinding.
**Tepper et al., 2022**	Surgery	eCOT-NS (external concurrent occipital and trigeminal neurostimulation)	Participants randomized to the sham group received an identical-appearing device, packaging, labeling, smartphone application, training, and treatment protocol as the active group. The sham device delivered symmetrical biphasic electrical waveforms (70–100 ms phase duration, 0.33 Hz) with peak output currents of up to 5 mA to the trigeminal branches and 10 mA to the occipital nerves for up to 60 minutes, mimicking the treatment experience while maintaining blinding.
**Niki et al., 2014**	Injection or nerve block	Lidocaine	Patients randomized to the placebo arm received 0.2 mL saline from an identical blinded metered-dose spray bottle. The investigator sprayed the solution onto the patient's palm, and the patient applied it with a finger to the painful oral mucosal area using the same application technique, dose (two 0.1-mL sprays), timing, observation period, and follow-up assessments as the lidocaine intervention.
**Mansano et al., 2022**	Surgery	Radiofrequency	Participants underwent the identical procedural protocol as the active radiofrequency group, including IV sedation (midazolam, fentanyl, and propofol), local anesthetic skin infiltration (2% lidocaine), fluoroscopic needle placement into Meckel's cave, and sensory/motor stimulation to confirm needle position. After confirmation, the needle was left in place for 60 seconds without delivery of thermal radiofrequency before removal; no local anesthetic was injected into Meckel's cave in any group.
**Reeve et al., 2024**	Injection or nerve block	BTA injection	Participants received intramuscular injections of 0.9% normal saline into the same five predefined masseter and temporalis muscle sites as the active treatment (1.5 mL per masseter and 0.5 mL per temporalis), using identical syringes, injection technique, needle size (27-gauge), and procedural protocol while awake to maintain blinding.
**Wu et al., 2012**	Injection or nerve block	BTA injection	Participants received 1.5 mL sterile isotonic saline injected at the same 15 intradermal and/or submucosal sites within the painful trigeminal dermatome (or oral mucosa when involved), using the same injection volume (0.1 mL/site), syringe, needle, and injection technique as the BTX-A group. Deeper muscle injections were avoided to prevent unwanted effects.

**Gazerani et al., 2008**	Injection or nerve block	BTA injection	Participants received subcutaneous isotonic saline injections on the contralateral (mirror) side of the forehead using the same injection technique, volume, anatomical location, and timing as the 22.5 U BoNT/A injections. Capsaicin was subsequently administered identically to both sides before and on days 1, 3, and 7 after treatment to induce pain and neurogenic inflammation under double-blind conditions.
**Zhang et al., 2014**	Injection or nerve block	BTA injection	Participants in the placebo group received identical intradermal and/or submucosal injections using the same number of injection sites and volume as the BTX-A groups, but with 1 ml saline solution (0.9%) instead of BTX-A. The injection technique and procedure were otherwise identical to maintain blinding.
**Wan et al., 2019**	Surgery	PRF (pulsed radiofrequency)	Participants underwent identical CT-guided Gasserian ganglion needle placement as the active PRF group; however, no radiofrequency energy was delivered. The procedure was otherwise identical to maintain blinding.
**Tjakkes et al., 2007**	Injection or nerve block	Ultracain	Participants received an intra-articular injection of sterile saline into the temporomandibular joint using the same injection technique and study procedures as the local anesthetic group. The study used a randomized, double-blind, placebo-controlled crossover design to maintain blinding.
**Bittar and Graff-Radford, 1993**	Injection or nerve block	Lidocaine	Participants received identical peripheral trigeminal nerve blocks with 2% lidocaine alone (3 mL), while the active group received 1 g streptomycin combined with 3 mL of 2% lidocaine. Injections were administered using the same intraoral technique (infraorbital or inferior alveolar nerve block) in a double-blind, randomized crossover design.
**Zúñiga et al., 2013**	Injection or nerve block	BTA injection	Participants received subcutaneous injections of 1 mL of 0.9% saline into the affected area. For patients with third division of the trigeminal nerve (V3) involvement, intramuscular placebo (0.9% saline) was also injected into the ipsilateral masseter muscle, matching the active BTX injection procedure to maintain blinding.

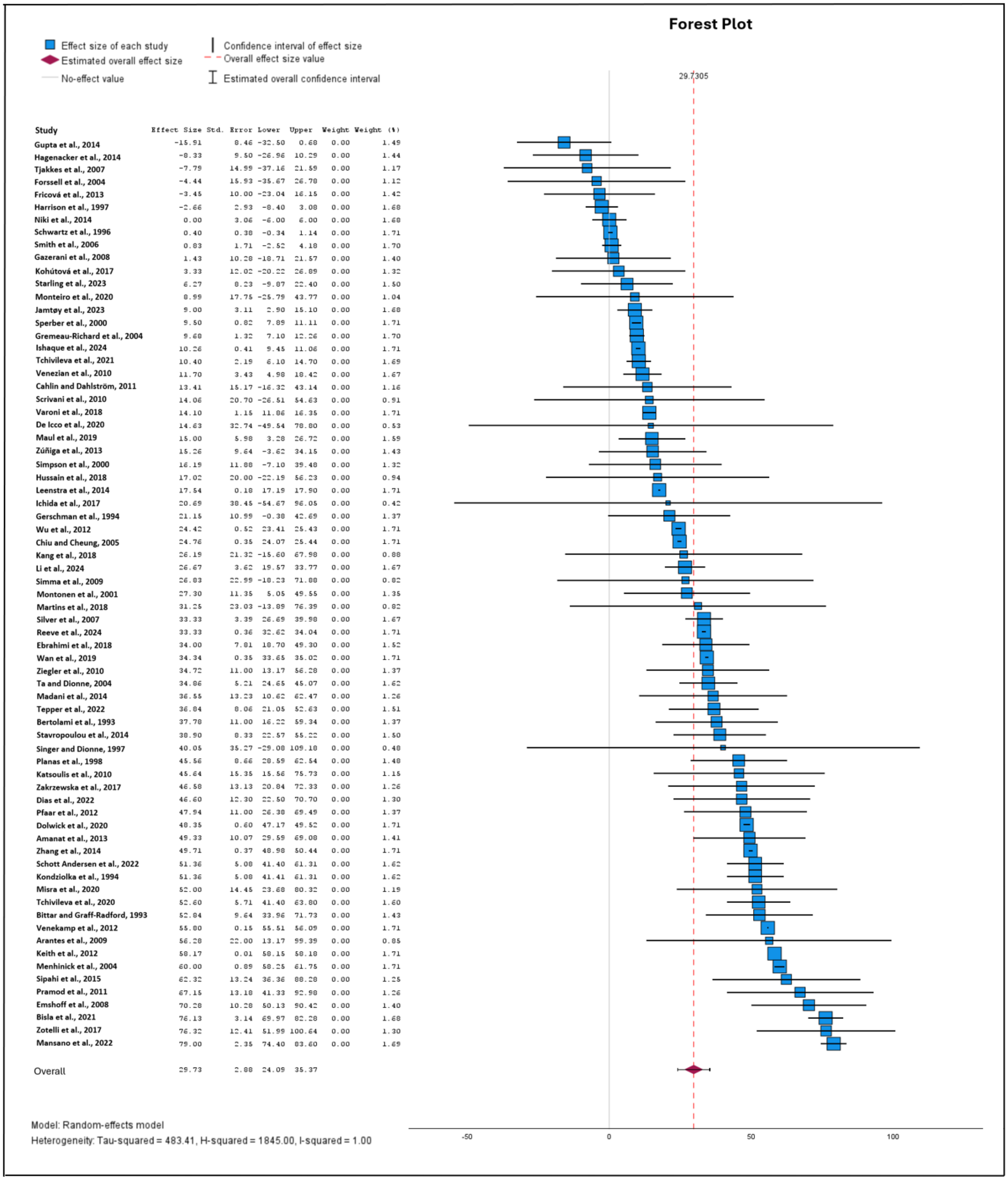

Figure 1 – **A)** Results of random effects analysis from 71 included studies in the estimation of percent improvement from facial pain treatments, using RStudio software and 'meta' library.
